# Structural insights into SetA-mediated Rab1 glucosylation and PI3P-guided localization during early *Legionella* infection

**DOI:** 10.1073/pnas.2535016123

**Published:** 2026-03-27

**Authors:** Ha Na Im, Yeon Lee, Yunju Song, Hyunggu Hahn, Hyerry Jeon, Donghyuk Shin, Sangho Lee, Kyung-Hee Kim, Kyung-Tae Kim, Se Won Suh, Dong Man Jang, Hyoun Sook Kim

**Affiliations:** ^a^Research Institute, National Cancer Center, Goyang 10408, Republic of Korea; ^b^Department of Chemistry, College of Natural Sciences, Seoul National University, Seoul 08826, Republic of Korea; ^c^Department of Biomedical Sciences, Seoul National University, Seoul 03080, Republic of Korea; ^d^Department of Systems Biology, College of Life Science and Biotechnology, Yonsei University, Seoul 03722, Republic of Korea; ^e^Department of Biological Sciences, Sungkyunkwan University, Suwon 16419, Republic of Korea; ^f^Department of Applied Chemistry, School of Science and Technology, Kookmin University, Seoul 02707, Republic of Korea; ^g^Department of Biological Chemistry and Molecular Pharmacology, Harvard Medical School, Boston, MA 02115

**Keywords:** *Legionella* infection, bacterial effector, SetA, crystal structures, Rab1

## Abstract

The bacterial pathogen *Legionella pneumophila* secretes effector proteins that remodel host endomembranes to establish a replication-permissive niche known as the *Legionella*-containing vacuole (LCV). Among these, SetA disrupts vesicle trafficking by glucosylating the small GTPase Rab1, essential for ER-to-Golgi transport. Here, we report comprehensive structural and mechanistic insights into SetA-mediated Rab1 glucosylation and its PI3P-dependent membrane targeting. Crystal structures of its N-terminal glycosyltransferase and C-terminal lipid-binding domains, captured in multiple ligand-bound states, reveal how SetA specifically recognizes GDP-bound Rab1 and the head group of phosphatidylinositol 3-phosphate (PI3P), which is enriched on early LCV membranes. SAXS-based full-length modeling, biochemical assays, and cellular imaging analyses demonstrate that SetA integrates Rab1 modification with membrane localization, thereby perturbing Golgi integrity and ER morphology. Together, these findings define the dual structural mechanisms underlying SetA’s coordination of substrate glucosylation and membrane association, providing a spatiotemporal framework for understanding *Legionella*’s early infection strategy.

## Significance

*Legionella pneumophila* remodels host membrane trafficking to establish a replication-permissive niche, yet the molecular basis of this process is not fully understood. Here, we elucidate the structural basis by which the effector SetA couples Rab1 glucosylation with PI3P-guided membrane localization during early *Legionella*-containing vacuole (LCV) maturation. By determining multiple structures of SetA and combining with biochemical and cellular analyses, we reveal how SetA selectively modifies GDP-bound Rab1 and targets PI3P-enriched membranes, thereby influencing early membrane remodeling events associated with LCV development. These findings uncover an integrated mechanism through which a single bacterial effector orchestrates both spatial positioning and enzymatic activity to modulate host organelle dynamics, providing an insight into early stages of *Legionella* infection.

Legionnaires’ disease is a severe and potentially fatal pneumonia that primarily affects the elderly and the immunocompromised. Its increasing prevalence, particularly in the industrialized world, remains a contemporary health concern (1, 2). *Legionella* is an environmental bacterium that parasitizes aquatic protozoans and replicates intracellularly. Long-standing coevolution with environmental protozoa has equipped *Legionella* with a diverse repertoire of virulence traits primarily optimized for replication within these natural hosts (3, 4). Consequently, human alveolar macrophages serve as permissive hosts for infection, as survival strategies evolved to counteract host cell-autonomous defense mechanisms in natural hosts remain effective against human cellular immunity. This highly adapted intracellular pathogen interferes with numerous host cellular functions, including vesicle trafficking, signal transduction, phospholipid metabolism, and inflammatory responses, ultimately subverting cellular immunity (5–7).

In particular, *Legionella pneumophila* establishes a replication-permissive membrane-bound niche, known as a *Legionella*-containing vacuole (LCV), which is orchestrated by the Dot/Icm type IV secretion system (T4SS) (8–10). This system translocates approximately 300 different “effector” proteins into host cells, constituting nearly 10% of the predicted protein-coding genes in several *L. pneumophila* strains (8–10). These effector proteins govern every step in the infection, LCV maturation, and replication processes by modulating membrane trafficking and hijacking the host cell machinery. To this end, some effectors possess domains for posttranslational modifications (ubiquitination, prenylation, phosphorylation, phosphocholination, methylation, AMPylation, and glycosylation) (6), targeting regulators of eukaryotic membrane dynamics such as phosphoinositides (PIs) and small GTPases (11). Notably, glycosylation plays a pivotal role in manipulating the cellular functions of key host signaling molecules, highlighting the increasing interest in glycosyltransferase toxins and effectors (12, 13). *Legionella g*lucosyl*t*ransferases (Lgts: Lgt1, Lgt2, and Lgt3) are representative glycosyltransferases (GTases) identified in *L. pneumophila*. Lgts selectively modify eukaryotic elongation factor 1A (eEF1A) by attaching a glucose (Glc) moiety using UDP-Glc (14, 15). The “glu”cosylation halts the elongation of nascent polypeptide chains, leading to severe toxicity in eukaryotic cells and indirectly affecting mTORC1 activation to obtain amino acids for bacterial consumption (16–18).

SetA (subversion of eukaryotic vesicle trafficking A; Lpg1978), identified as another *Legionella* glucosyltransferase, emerged from a phenotypic screen of *L. pneumophila* effectors lethal to yeast, disrupting eukaryotic vesicle trafficking and affecting growth and secretory functions (19). Although a SetA-deficient *L. pneumophila* strain displayed unaltered replication in murine bone marrow–derived macrophages and *Dictyostelium discoideum* (19), ectopic expression of SetA exhibited toxic effects on yeast and certain mammalian cell lines, such as HEK293T and HeLa cells (20). This ability of SetA is linked to its GTase domain containing 300 N-terminal amino acids that transfer the Glc moiety via UDP-Glc (19, 20). SetA, which is present in all sequenced pathogenic *L. pneumophila* strains, possesses a conserved key motif akin to typical *Legionella* GTases (Lgt1-3). However, SetA diverges by glycosylating the small GTPase Rab1 at Thr75 within the switch II region, whereas Lgt1-3 target eEF1A to inhibit protein translation (21). Modifications in Rab1, such as glycosylation, hold significant importance in LCV maturation, as Rab1 orchestrates vesicular transport between the endoplasmic reticulum (ER) and the Golgi apparatus (22). Interestingly, SetA prefers to modify GDP-bound Rab1, resulting in decreased affinity of Rab1 with a GDP disassociation inhibitor (GDI); however, it does not affect SidM, the guanine nucleotide exchange factor of Rab1. This modification inhibits the intrinsic GTPase activity of Rab1, elevating GTP-bound Rab1 levels and enabling multiple Dot/Icm effectors to access GTP-bound Rab1 (21). SetA also modifies various eukaryotic proteins, indicating its potential as a universal *O*-glucosylation system for protein engineering (23, 24) and underscoring the need to comprehend the substrate specificity of SetA at the molecular level.

Together with LCV-decorated small GTPases, such as Rab1, specific LCV phosphoinositide patterns define the progression of LCV formation and maturation during each stage, which are also governed by the spatiotemporal arrangement of *Legionella* effectors (25, 26). Of particular importance in early LCV formation is the acquisition of phosphatidylinositol-3-phosphate (PI3P), which is then gradually cleared from LCVs to facilitate the enrichment of phosphatidylinositol-4-phosphate (PI4P) on the surface of the LCV that functions as an ER-like replication compartment (27). A previous study highlighted the distinct role of the C-terminal region of SetA (amino acids 401 to 644) in specific binding to PI3P, which is abundant in early endosomal membranes (20). Given that the N-terminal GTase domain and the C-terminal region of SetA contribute significantly to a severe growth defect (20), it is intriguing that the PI3P-binding ability of SetA is responsible for its cellular function.

This study aimed to unravel the structural and molecular mechanisms underlying Rab1 glucosylation and recognition of various ligands, including PI3P, by *L. pneumophila SetA*. To this end, we determined the crystal structures of the N-terminal GTase domain, the C-terminal lipid-binding domain, and their complexes with the GTase donor substrate (UDP·Glc) and product (UDP), as well as the head moiety of PI3P. Complemented by biochemical, biophysical, in silico docking with Rab1, and small-angle X-ray scattering (SAXS) studies, these comprehensive snapshots of SetA complexes in multiple states provide insights into how SetA specifically recognizes GDP-bound Rab1 and PI3P during the early stage of *Legionella* infection and how its multidomains work in concert. These findings serve as a foundation for understanding the strategies of *Legionella* to evade rapid endocytic maturation in a spatiotemporal manner.

## Results

### Cytotoxic Impact and Overall Structural Envelope of the SetA Effector.

**Fig. 1.**
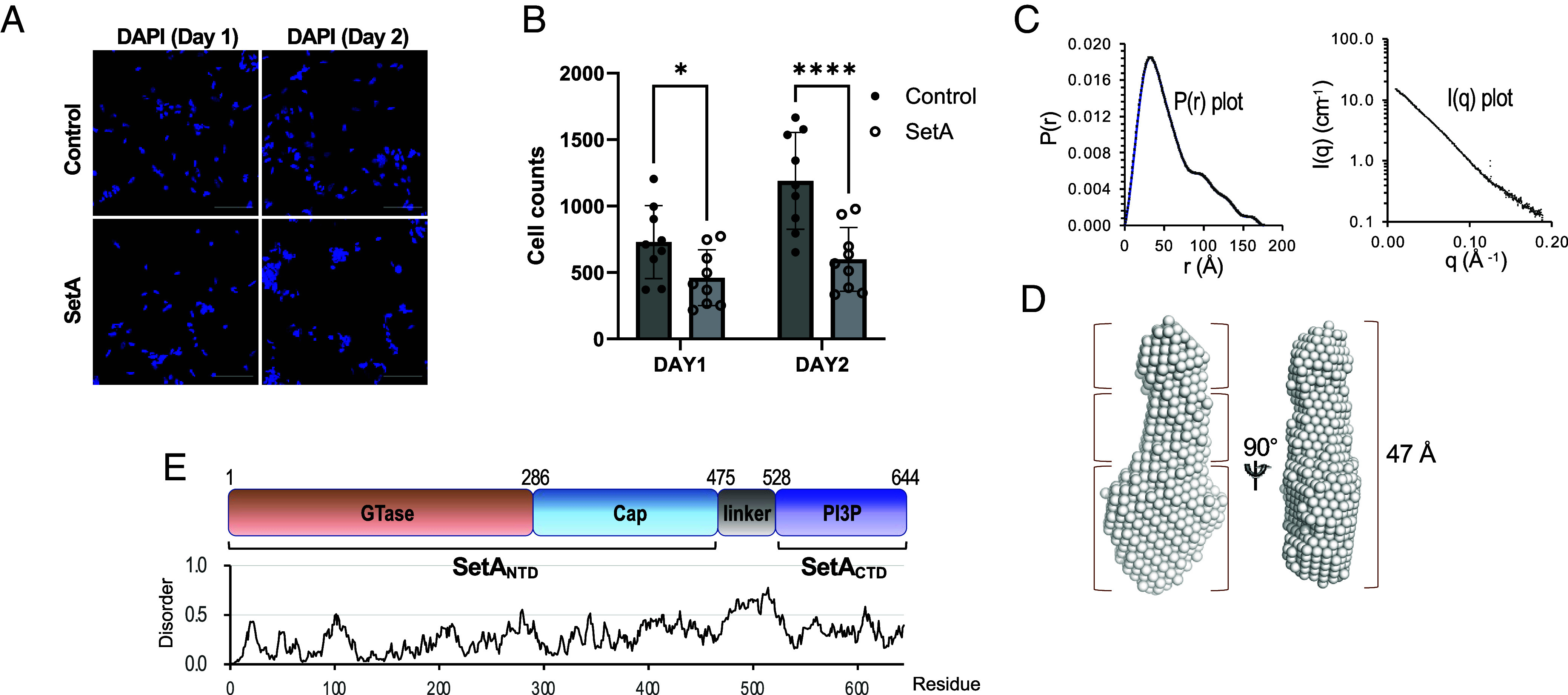
Cytotoxic impact and overall structure analysis of *Legionella pneumophila* SetA in solution. (*A*) Microscopic analysis of HEK293 cells upon SetA overexpression. DAPI-stained nuclei were imaged at day 1 and day 2 after transfection. (Scale bar, 100 µm.) (*B*) Quantification of cell counts based on DAPI staining at day 1 and day 2. Data are presented as mean ± SD (n = 9); *P* < 0.05, ***P < 0.0001 (unpaired *t* test). (*C* and *D*) Small-angle X-ray scattering (SAXS) analysis of SetA. (*C*) The *Left* panel shows pair distribution function [P(r)], with a maximum dimension of approximately 150 Å. The *Right* panel displays the scattering intensity [I(q)] as a function of momentum transfer (q), fitted to the experimental SAXS data. (*D*) The molecular envelope of SetA in two views, indicating an elongated shape. (*E*) Domain architecture of SetA. SetA consists of a glycosyltransferase (GTase) domain (residues 1 to 285), a cap domain (residues 286 to 474), a flexible linker (residues 475 to 527), and a PI3P-binding domain (residues 528 to 644). The plot below shows predicted disorder regions, with higher values indicating increased flexibility.

To initiate our investigation into the potential biological relevance of the SetA effector, particularly in the context of its potential cytotoxicity during infection, we assessed the proliferation of HEK293 cells upon SetA overexpression. Microscopic analysis of DAPI-stained nuclei revealed a pronounced reduction in cell number (Fig. 1 *A* and *B*). While these elevated expression levels may not fully recapitulate physiological conditions during infection, the observed impact suggests that SetA has the potential to perturb host cellular pathways regulating proliferation and survival under conditions of its elevated expression. To connect structural attributes with the observed cellular effects, we initially examined the overall envelope and conformation of SetA in solution using SAXS analysis. Due to the polydispersity and instability of the full-length SetA (1 to 644; SetA_full_) during SAXS analysis, we utilized a more stable variant, SetA_full∆13_. This construct was generated by truncating the N-terminal 13 disordered residues (Fig. 1*E*) and introducing K577A/K578A/K580A mutations based on the Surface Entropy Reduction (SER) approach (28) (*SI Appendix*, Table S1). SAXS analysis of SetA_full∆13_ at multiple concentrations revealed that SetA_full∆13_ does not undergo concentration-dependent aggregation or oligomerization, and the calculated radius of gyration (Rg) of SetA_full∆13_ at the highest concentration was 47.43 Å (Fig. 1*C*). The calculated molecular weight (72.6 kDa) determined from the SAXS data closely matched the theoretical value (72.3 kDa), as independently validated by SEC-MALS (*SI Appendix*, Fig. S1). These data indicate SetA_full∆13_ exist predominantly as a monomer in solution.

The *ab initio* SAXS envelope revealed an elongated, arrow-like structure with discernible domain features: a large, flat, and wide domain at the arrowhead, a compact globular domain at the tail, and an extended middle region (Fig. 1*D*). To assign these structure features, we performed disorder prediction and domain mapping based on sequence features and SAXS volume constraints, which allowed precise identification of intrinsically disordered regions and functional domains (Fig. 1*E*). These analyses guided the design of multiple constructs suitable for crystallographic studies, facilitating structural integration with the SAXS-derived SetA_full∆13_ structure, as detailed further in the following sections.

### SetA_NTD_ Structure Reveals a Two-Subdomain Architecture.

**Fig. 2.**
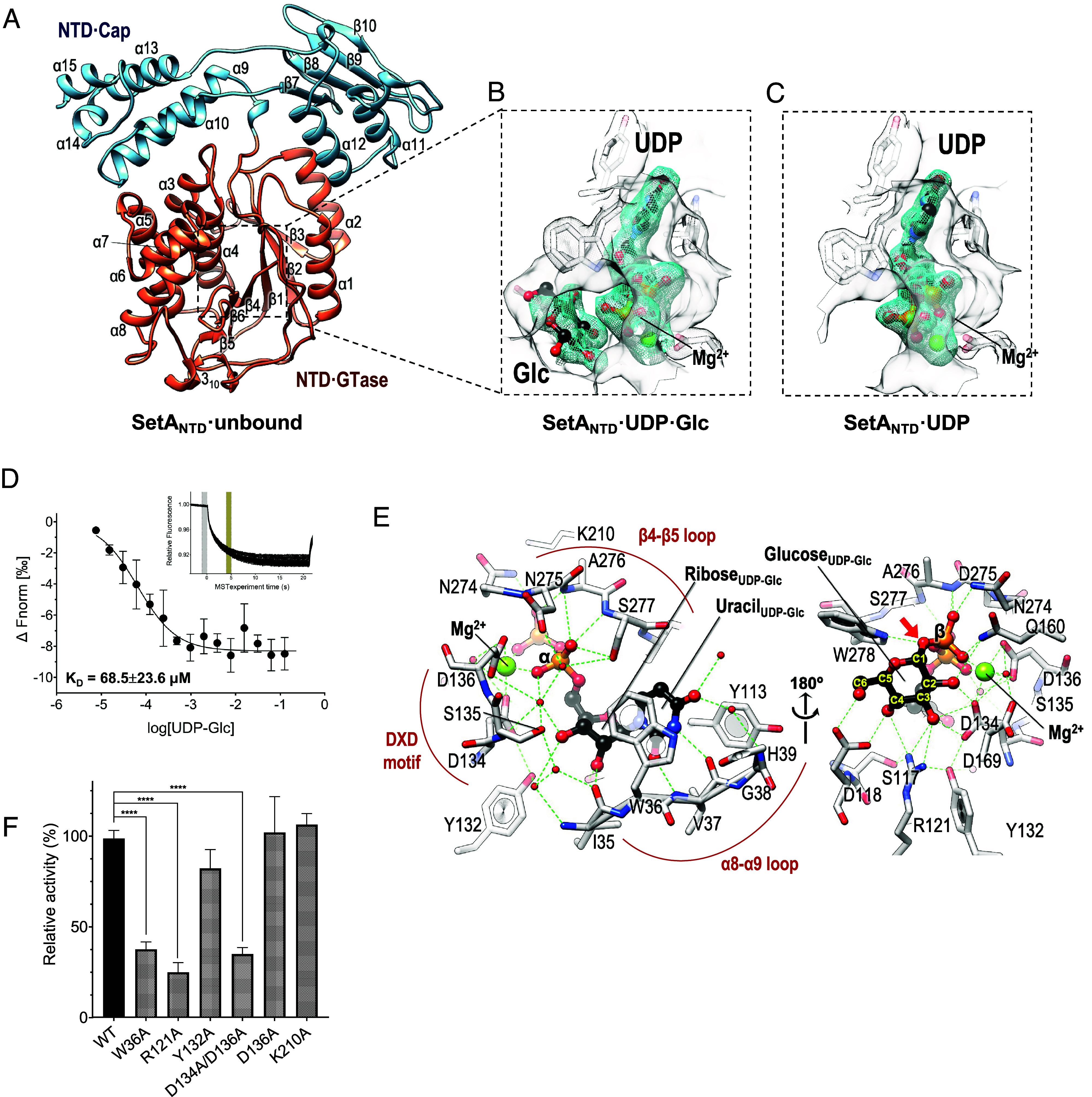
Structural and functional characterization of SetA_NTD_ in the ligand-bound and -unbound states. (*A*) Overall structure of SetA_NTD·unbound_ showing the GTase (orange) and Cap (cyan) subdomains. Secondary structures are displayed as cartoon representations. (*B* and *C*) Close-up views of the SetA_NTD_ structures in complex with UDP·Glc (SetA_NTD_·UDP·Glc) (*B*) or UDP (SetA_NTD_·UDP) (*C*), showing the *mFo*–*DFc* omit electron density maps (cyan mesh, contoured at 2.0 σ) for the SetA_NTD_-bound UDP·Glc (*B*) and UDP (*C*) within the SetA_NTD_ active-site pocket indicated by a gray surface. Note that UDP·Glc is modeled based on the separate UDP and glucose molecules observed in the electron density map. UDP·Glc or UDP and Mg^2+^ are shown as stick and ball representations, respectively. (*D*) Binding affinity of SetA_NTD_ for UDP-Glc as measured using microscale thermophoresis (MST). The inset graph indicates the MST trace. The dissociation constant (*K_D_*) value represents the means ± SD of three independent experiments. (*E*) Close-up view of the UDP–Glc binding site. Residues from the β4–β5 and α8–α9 loops form hydrogen-bonds and π–π interaction network that locks the phosphate and uracil moieties of UDP–Glc. The α- and β-phosphate groups are stabilized by Ser135, Asn274, Ala276, and Ser277, whereas the uracil base is coordinated through hydrogen bonding with Val37 and His39 (via a water molecule) and π-stacking with Trp36 and Tyr113. Ribose hydroxyl groups additionally interact with Ile35, Ser117, Tyr132, and the Leu133–Ser135 backbone. (*F*) Mutational analysis of key residues in the active site. Relative UDP-Glc hydrolysis activities of SetA mutants compared to wild-type (WT) SetA. The W36A, R121A, Y132A, and D134A/D136A mutants showed significantly reduced enzymatic activity. Error bars represent the mean ± SD from three independent experiments (****P < 0.0001).

In order to clarify the precise structural mechanism behind SetA-mediated glucosylation, we first determined the crystal structure of its N-terminal structured region (Met14–Gly474; SetA_NTD_·unbound) in the absence of any ligand at a 2.5 Å resolution using the single-wavelength anomalous diffraction (SAD) method (Fig. 2*A* and *SI Appendix*, Table S2). This region encompasses 300 N-terminal residues of SetA, which were previously demonstrated to possess sufficient GTase enzymatic activity, and were thus expected to harbor a GTase (20). Interestingly, the SetA_NTD_ structure displayed an inverted-triangle shape comprising two subdomains, a GTase core domain (SetA_NTD·GTase_; residues 26 to 286) and a core-associated subdomain (SetA_NTD·Cap_; residues 287 to 474) (Fig. 2*A*). The SetA_NTD·GTase_ structure adopts the fold of the central six-stranded β-sheet (β3↑-β2↑-β1↑-β4↑-β6↓-β5↑) flanked by two bundles of α-helices (α1 and α2; α3–α8), which cover the β-sheet from the sides along with several short 3_10_ helices (Fig. 2*A*). The SetA_NTD·Cap_ consists of two parts, five alpha helices (α9–α10 and α13–α15) and the α/β region (β7↓-β8↓-β9↑-β10↓ and α11–α12) (Fig. 2*A*). Given that the interface area the two subdomains share is 1,057 Å^2^ (approximately 9% of the surface area of SetA_NTD·GTase_) and the interface is primarily lined with various hydrophobic residues (such as Ile40, Ile46, Leu77, Tyr191, Ile192, Leu302, Phe305, Ala406, and Leu409) (*SI Appendix*, Fig. S2), the domain configuration shown in the SetA_NTD_ structure is remarkably favorable. In particular, the SetA_NTD·Cap_ subdomain shields the hydrophobic surface of SetA_NTD·GTase_ from the solvent and securely holds the top of the GTase subdomain like a cap, connecting the SetA_NTD·GTase_ core domain with the subsequent C-terminal domains of SetA (Fig. 1*E*).

### Donor Substrate- and Product-Bound States of SetA_NTD_.

As a GTase, SetA utilizes UDP-Glc as a donor substrate, with a dissociation constant (*K*_D_) of 68.5 ± 23.6 μM determined from microscale thermophoresis (MST) experiments (Fig. 2*D*). To understand the structural determinants for UDP-Glc binding, we next determined the crystal structures of SetA_NTD_ in complex with UDP·Glc (SetA_NTD_·UDP·Glc) as a donor substrate (Fig. 2*B*) or UDP (SetA_NTD_·UDP) as a product (Fig. 2*C*). In the structure of SetA_NTD_·UDP·Glc, the disconnected extra electron densities were modeled as separate UDP and glucose molecules (Fig. 2*B*), reflecting a hydrolyzed donor state resulting from transfer of the glucosyl moiety to a water molecule during crystallization. While the covalent linkage is cleaved, the UDP and glucose molecules remain bound in their respective sites, preserving a configuration nearly identical to the donor substrate. Because this arrangement captures the binding mode prior to product dissociation, we hereafter refer to this hydrolyzed state as the “substrate-bound state” for simplicity.

The UDP·Glc molecule is ensconced within the pocket in the SetA_NTD_·UDP·Glc structure, indicating the donor substrate site in SetA_NTD_ (Fig. 2*B*). This reveals tight interactions between the UDP·Glc molecule, several key residues of SetA_NTD_, and the divalent magnesium cation (Fig. 2*E*). The magnesium cation interacts with UDP diphosphate, the Asp134^Oδ1^ and Asp136^Oδ1,Oδ2^ residues constituting the typical DXD motif conserved across the GTase family, and an ordered water molecule in an octahedral fashion (Fig. 2*E*). Numerous residues on the β4-β5 and α8-α9 loops participate in interactions that tightly lock the phosphate groups of UDP (Fig. 2*E*).

The overall structures of the donor substrate- and product-bound SetA_NTD_ were nearly identical, with the UDP molecule bound in the same manner (Fig. 2 *B* and *C*). However, in the structure of SetA_NTD_·UDP·Glc, the glucose moiety is stabilized by the electrostatic and hydrophobic interactions involving Trp278^Nε2^ and hydrogen-bonding with Asp118^Oδ1,Oδ2^, Arg121^Nη1,Nη2^, Asp134^Oδ1,Oδ2^, and Gln160^Nε2^ (Fig. 2*E*). Conversely, in the structure of SetA_NTD_·UDP, four water molecules occupy the position corresponding to glucose (figure not shown). The C2 hydroxyl group of glucose is particularly noteworthy as it forms hydrogen bonds with Asp134^Oδ2^ and Gln160^Nε2^, which may contribute to the preferential binding of SetA to UDP-Glc over UDP-GlcNAc (Fig. 2*E*). Mutagenesis studies focusing on key residues involved in UDP·Glc binding demonstrated that Trp36, Arg121, and Asp134 were essential for GTase activity (Fig. 2*F*).

### Conformational Changes of Active Site Loops Upon UDP·Glc Binding.

**Fig. 3.**
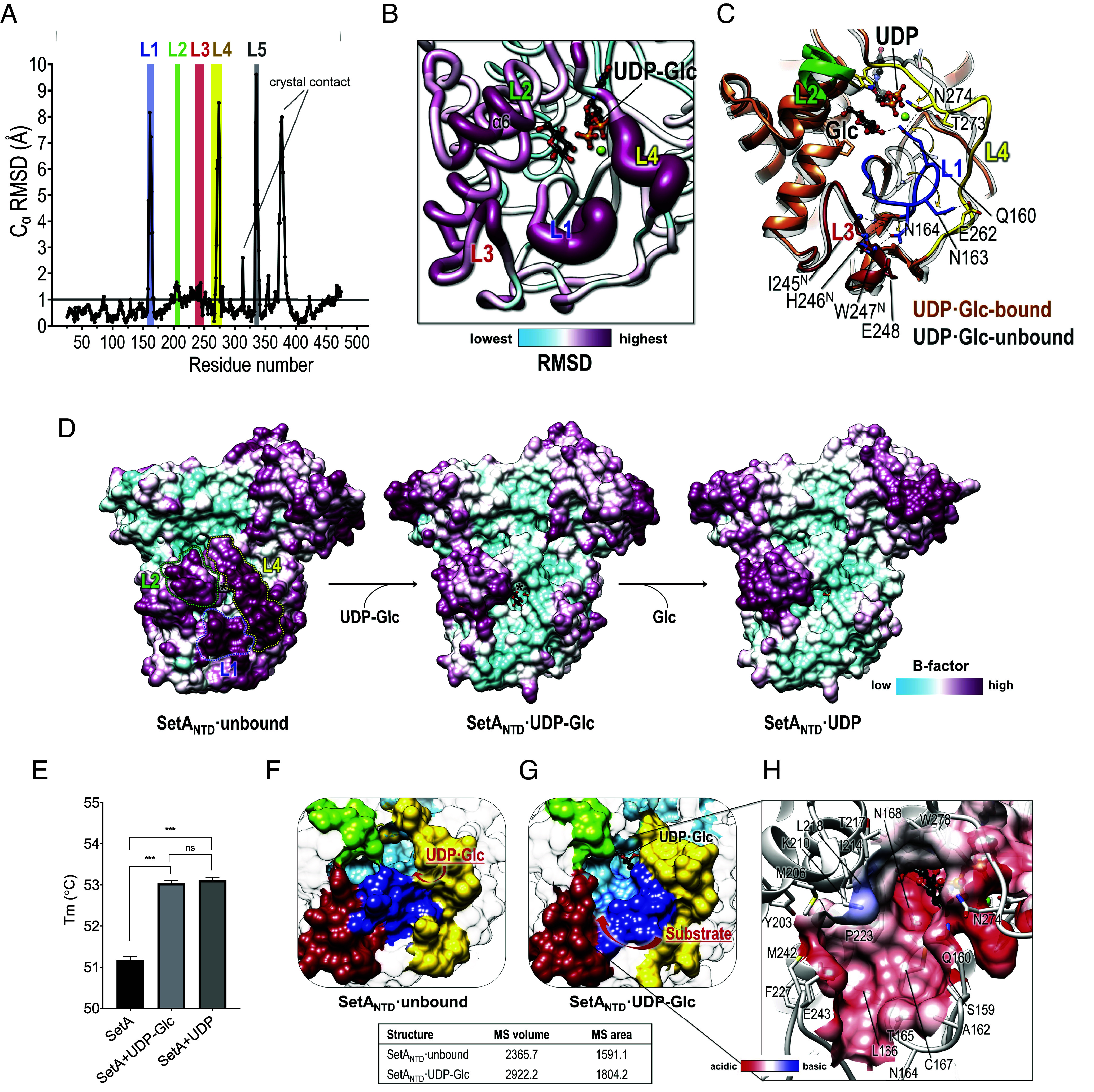
Conformational changes of SetA_NTD_ upon UDP·glucose or UDP binding. (*A*) Plot of the C_α_ Rms deviations (RMSDs) between the structures of SetA_NTD·unbound_ and SetA_NTD·UDP·Glc_. The L1–L5 regions (colored bars) display increased RMSDs, indicating conformational changes. The subtle changes in the crystal contact loop regions are indicated by lines. (*B*) Close-up view of the SetA_NTD_ active site, highlighting the L1–L4 regions with the most significant structural deviations (thickened and colored by RMSD scale) when transitioning from the unbound to UDP·Glc-bound states. The UDP·Glc molecule is shown in a stick representation. (*C*) Superposition of the structure of SetA_NTD_ in its unbound (gray) and UDP·Glc-bound (colored) states, showing the key conformational changes in the loops. Active site residues involved in significant shifts upon ligand-binding are labeled and displayed as stick representations. (*D*) Surface representations of the structures of SetA_NTD_ in the unbound (*Left*), UDP·Glc-bound (middle), and UDP-bound (*Right*) states, colored by B-factor (sky blue to purple). The binding of UDP·Glc and UDP induces localized rigidification, particularly in L1, L4, and the central substrate-binding groove. (*E*) Thermostability comparison of SetA_NTD_ in different states as measured using differential scanning fluorimetry. SetA_NTD_ exhibits significantly higher melting temperature (Tm) in the UDP·Glc-bound and UDP-bound states compared to the unbound state. Error bars represent the mean ± SD from three independent experiments (***P < 0.001; ns, not significant). (*F* and *G*) Molecular surface volume (Å^3^) and area (Å^2^) calculations for SetA_NTD·unbound_ (*F*) and the SetA_NTD·UDP·Glc_ complex (*G*), showing an increase in both volume and surface area upon UDP·Glc binding. (*H*) Electrostatic surface representation of the potential binding cavity for the acceptor substrate in SetA_NTD·UDP·Glc_, highlighting the negatively charged elongated groove formed after the UDP·Glc association and the subsequent conformational changes. Key residues lining the active site cavity are labeled and shown in a stick representation.

Although the overall structure of SetA_NTD_·UDP·Glc remained essentially similar to that of UDP-Glc-free SetA_NTD_ with an RMSD of 1.03 Å over 449 equivalent C_α_ positions for residues of Ala26–Gly474, SetA_NTD_·UDP·Glc exhibited significant conformational changes on four loop regions around the UDP-bound active site: L1 (residues 157 to 168) in the β5-β6 loop, L2 (residues 202 to 209) in the α6-α7 loop, L3 (residues 235 to 250), and L4 (residues 261 to 279) in the α8-α9 loop kinked by the presence of 3_10_ helices (Fig. 3 *A* and *B*). Drastic conformational changes are evident in L4, with the largest C_α_-RMSD of 2.55 Å. This is exemplified by the significant shift (8.53 Å) observed in Asn274^Cα^ in this region, enabling it to adopt a conformation suitable for UDP·Glc binding (Fig. 3*C*). L4 can also be repositioned closer to the nucleotide-binding site spanning the GTase core surface. This repositioning of L4 concomitantly induces concerted rearrangements of L1 and L3 through hydrogen-bonding networks among the loops. In detail, the Asn274 in L4 interacting with the UDP is in position to H-bond with Gln160 of L1, which undergoes a large shift of 6.58 Å (Fig. 3*C*). The L1 loop then rotates by approximately 90°, allowing Asn163 and Asn164 to establish hydrogen bonds with residues in the L3 and L4 loops (Fig. 3*C*), causing cooperative changes in their conformations. A comparison of the B-factor distributions for the structures of SetA_NTD_ unbound, SetA_NTD_ UDP·Glc, and SetA_NTD_ UDP showed that the active site loops in the nucleotide-free state were flexible (Fig. 3*D*). However, with the addition of UDP·Glc or even UDP binding, the regions of the L1 and L4 loops were stabilized with lower B-factors (Fig. 3*D*). Indeed, the measurement of melting temperatures supported the idea that thermal stability was significantly increased in UDP·Glc- or UDP-bound SetA_NTD_ compared to the unbound form (Fig. 3*E*).

One effect of such conformational alterations of the specific loops upon UDP·Glc binding is the enlargement of the solvent-accessible surface area within the active site cavity, depicted as a light-blue surface in Fig. 3 *F* and *G*. In the absence of nucleotide binding, the active site cavity was partially obstructed by the positioning of L1 to L3, offering a restricted space for the binding of a divalent cation and UDP·Glc (Fig. 3*F*). In contrast, after UDP·Glc association and the subsequent displacement of L1 to L4 within the SetA_NTD_ UDP·Glc structure, an elongated groove in a widened and opened form was observed, where considerably more residues were uncovered on the cavity surface (Fig. 3 *G* and *H*). The electrostatic representation of the cavity revealed a lining of hydrophobic residues (Ala162, Thr165, Leu166, Leu218, Pro223, Ala244, and Ile245) along with an acidic residue, such as Glu243 (Fig. 3*H*). The increase in the solvent-accessible surface area of the active site cavity in the SetA_NTD_ UDP·Glc structure seems predisposed to the engagement of acceptor substrates, thereby offering a specific path for acceptor substrate binding as well as its departure after the enzymatic sugar-transferring reaction.

### SetA_NTD_ Specifically Recognizes the Rab1 *O*-Glucosylation Motif.

**Fig. 4.**
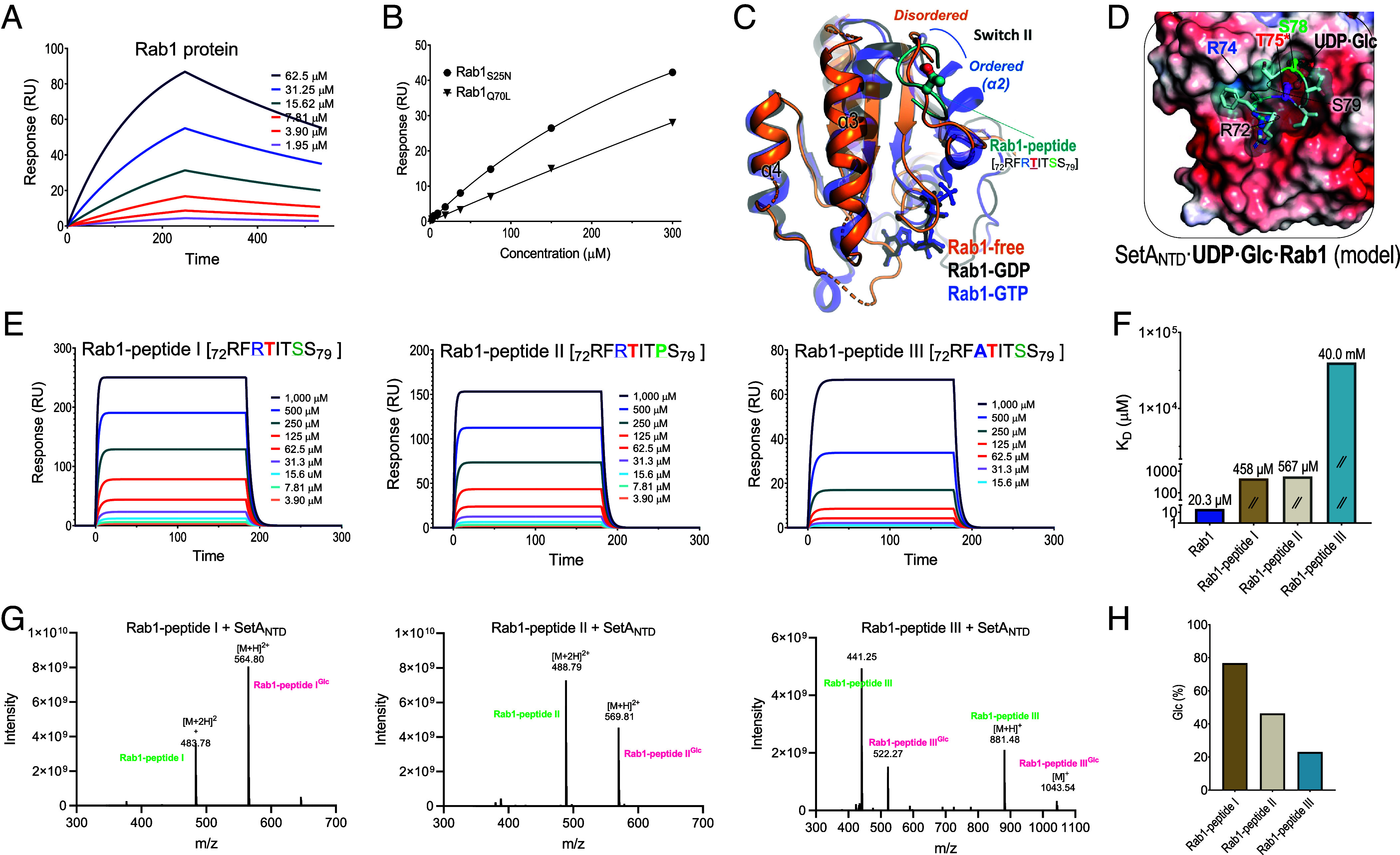
Rab1 binding and glucosylation by SetA_NTD_. (*A*) Surface plasmon resonance (SPR) analysis of Rab1 binding to SetA_NTD_. The SPR sensorgram displays the binding of Rab1 proteins to immobilized SetA_NTD_ proteins at increasing concentrations. (*B*) SPR analysis of Rab1_Q70L_ (GTP-bound mimic) and Rab1_S25N_ (GDP-bound mimic) binding to SetA_NTD_. The equilibrium responses, recorded 230 s after Rab1 injection, were plotted as a function of Rab1 protein concentration. (*C*) Structural comparison of ligand-free, GDP-bound, and GTP-bound Rab1 (PDB IDs: 3L0I, 2FOL, and 3TKL, respectively). The GTP-bound Rab1 structure (purplish blue) exhibits an ordered helix (α2) in the switch II region, while the ligand-free (orange) or GDP-bound (black) Rab1 structures show a loop or disordered conformation. The Rab1-derived peptide (cyan) containing Thr75 (^72^RFRTITSS^79^), which was docked into the structure of SetA_NTD·UDP·Glc_, is superposed onto the ligand-free Rab1 structure. (*D*) Docking model of the Rab1 peptide against the SetA_NTD·UDP·Glc_ structure. The SetA_NTD·UDP·Glc_ structure is displayed in an electrostatic surface representation. The Rab1 peptide, depicted in stick and cartoon representations, is nestled within the active site cavity formed after UDP·Glc binding, with the critical residues R72, T75, and S79 making key interactions. (*E*) SPR analysis of Rab1-derived peptides binding to SetA_NTD_. Three Rab1-peptides I (^72^RFRTITSS^79^), II (^72^RFRTITPS^79^), and III (^72^RFATITSS^79^) were tested at concentrations ranging from 3.90 to 1,000 μM. (*F*) Dissociation constants (K_D_) for Rab1 proteins and Rab1-peptides I–III binding to SetA_NTD_. (*G*) Mass spectrometry analysis of glucosylation of the Rab1-peptides by SetA_NTD_. The spectra show the addition of a glucosyl group (+81 or +162 Da) on Rab1-peptides I–III, confirming SetA_NTD_-mediated glucosylation. (*H*) Quantification of the conversion rate of Rab1-peptides glucosylation by SetA, based on mass spectrometry results.

Since a recent study demonstrated that Rab1 as a specific substrate is α-*O*-glucosylated by SetA during *L. pneumophila* infection to inhibit GTPase activity (21), we aimed to further elucidate how SetA recognizes Rab1 and clarify the specific recognition motif involved. First, we confirmed that Rab1 proteins or a synthetic Rab1 peptide (^73^FRTITSSYYR^82^) were glucosylated by SetA_NTD_ at the Thr75 residue using mass analysis (*SI Appendix*, Figs. S3 and S4). The surface plasmon resonance (SPR) experiments with the recombinant Rab1 proteins gave a dissociation constant of 20.3 ± 0.5 μM (Fig. 4*A*).

Since previous work has highlighted that SetA preferentially modifies the GDP-bound form of Rab1 (21), we next compared the binding affinities of two mutants Rab1_S25N_ and Rab1_Q70L_, mimicking the GDP- and GTP-bound states, respectively. The GDP-bound mutant bound SetA_NTD_ with a dissociation constant of 15.7 μM, approximately 60-fold stronger than the GTP-bound form (*K*_D_ = 0.94 mM) (Fig. 4*B*). A closer inspection of Rab1 structures showed that the sequence surrounding the catalytic Thr75 of Rab1 was characterized by a highly conserved switch II region of small GTPases. This region is typically flexible or disordered in the GDP-bound state but forms the α2 helix in the GTP-bound state (Fig. 4*C*). Notably, our SetA_NTD_·UDP·Glc structure reveals the characteristic cavity for an acceptor substrate (Fig. 3*H*), however insufficient to accommodate the α2 helix of the GTP-bound Rab1. Together with the binding affinity result, SetA is thought to recognize a flexible form of the switch II loop that includes Thr75 of GDP-bound Rab1. To explore the interaction mode between both proteins, we performed computational Glide docking (29) using the Rab1 peptide (^72^RFRTITSS^79^), a part of the switch II in the GDP-bound state, against the SetA_NTD_·UDP·Glc structure. The best-scoring pose showed that the deep cavity of SetA_NTD_ can accommodate Rab1 Phe73 and Ser78, with the side chain hydroxyl group of Thr75 positioned near the glucose molecule bound to SetA_NTD_ and the side chain of Arg74 located within the acidic region of the cavity (Fig. 4*D*).

Previous proteomic profiling revealed that SetA can introduce *O*-glucose modifications to various eukaryotic proteins with the favored sequence motif (S/T)xxL(P/G) (23). Since the Rab1 sequence appeared to deviate from the proposed motif, we performed a series of SPR experiments with Rab1-derived peptides to determine whether SetA_NTD_ could selectively recognize the Rab1 sequence over the motif. First, the original Rab1 peptide (^72^RFRTITSS^79^; Rab1 peptide I) had a dissociation constant of 458±4 μM, which is ~22-fold weaker compared to the endogenous Rab1 protein (Fig. 4 *E* and *F*). This implies that the Arg72–Ser79 residues of Rab1 are necessary for catalysis by SetA but are insufficient for robust binding. Substituting Ser78 with Pro at the *i*+3 position (^72^RFRTIT**P**S^79^; Rab1 peptide II) slightly weakened affinity (*K*_D_ = 567 ± 5 μM) (Fig. 4 *E* and *F*). Surprisingly, substituting Arg74 to Ala at the *i*−1 position (^72^RF**A**TITSS^79^; Rab1 peptide III) drastically reduced binding to SetA_NTD_ (*K*_D_ = 40 ± 3 mM), ~87-fold weaker than the original peptide (Fig. 4 *E* and *F*).

Consistently, limited enzymatic reactions and mass shift analysis showed that peptides I–III were glucosylated with conversion rates of 76.7%, 46.3%, and 23.0%, respectively (Fig. 4 *G* and *H* and *SI Appendix*, Fig. S4). Therefore, Rab1 Arg74 is crucial not only for binding to the SetA_NTD_ cavity but also for improving the catalytic performance of SetA. This also supports the Rab1 peptide-docking model in which Arg74 is concealed on the surface of SetA_NTD_ with multiple hydrogen bonds (Fig. 4*D*). Taken together, these findings imply that SetA_NTD_ can discern the catalytic sequence of Rab1 in a specific manner, reinforcing the physiological role of SetA in modulating Rab1 on early endosomes following *Legionella* infection.

### Structural Determinants of SetA_CTD_ for Recognizing PI3P.

**Fig. 5.**
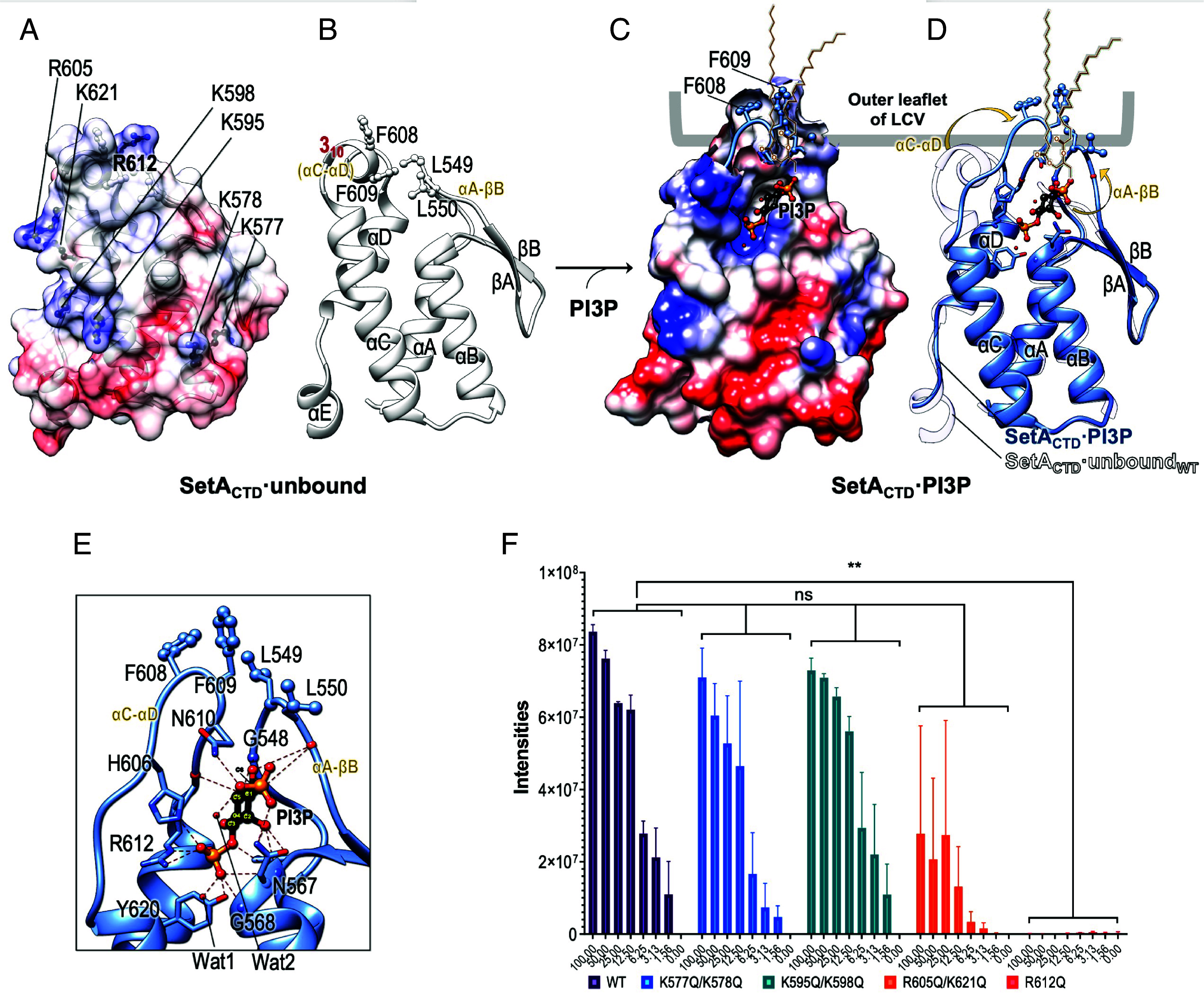
Structural insights into PI3P recognition by SetA_CTD_. (*A*) Electrostatic surface representation of SetA_CTD·unbound_, highlighting the positively charged residues (K577, K578, K595, K598, R605, R612, and K621) in blue. (*B*) Ribbon representation of SetA_CTD·unbound_, showing the positions and conformations of the key residues F608, F609, L549, and L550 displayed in a stick representation. (*C*) Electrostatic surface representation of the SetA_CTD·PI3P_ complex, showing the bound PI3P head group (shown in a stick model) in the positively charged pocket. The bound ligand, F608, and F609 residues are depicted in a stick representation. The outer leaflet of the *Legionella*-containing vacuole (LCV) and the aliphatic lipid acyl chains potentially linked to the PI3P head group are shown. (*D*) Structural comparison of SetA_CTD·PI3P_ (blue) and SetA_CTD·unbound_ (white), highlighting the structural rearrangement of the αC-αD and αA-βB loops upon PI3P binding, which may contribute to its anchoring into the LCV outer leaflet. (*E*) Detailed view of the PI3P-binding pocket in SetA_CTD·PI3P_. The PI3P head group and key interacting residues are shown in a stick representation, while water molecules (Wat1 and Wat2) and the main-chain nitrogen or oxygen molecules are depicted as balls. Hydrogen bonds are indicated by dotted lines. (*F*) Mutational analysis of SetA_CTD_ using a lipid-binding assay, showing the effects of specific mutations on PI3P binding. The bars represent the binding intensities of the WT and mutant SetA_CTD_ proteins to PI3P, measured at various amounts (1.56 to 100 pmol). The R612Q mutation exhibited the most significant reduction in binding affinity compared to the WT and other mutants. Error bars represent the mean ± SD from three independent experiments (**P < 0.01).

Having defined the substrate specificity and structural features of SetA_NTD_, we next focused on the membrane binding module of SetA. Previous studies showed that SetA localizes to the LCV membrane through PI3P recognition by its C-terminal region (20). Our SAXS data on SetA_full∆13_ revealed another small globular domain at the C-terminus, likely corresponding to the PI3P-binding domain (Fig. 1*D*). We first confirmed that SetA_full_ or the SetA C-terminal domain (SetA_CTD_: residues 528 to 644) specifically interacts with PI3P using protein–lipid overlay assays (*SI Appendix*, Figs. S5 and S6). To provide a structural basis for the direct interaction between PI3P and SetA, we next determined the crystal structures of SetA_CTD_, in complex with inositol 1,3-bisphosphate [Ins(1,3)P2], a head group of PI3P, at 2.45 Å resolution using the SAD method (Fig. 5 *A–D* and *SI Appendix*, Table S2). Due to crystal lattice contacts that restricted access to the binding site in one molecule, only one of two SetA_CTD_ molecules in the asymmetric unit established a complex with the PI3P headgroup (SetA_CTD_·PI3P) (Fig. 5 *C* and *D*), while the second remained unbound (SetA_CTD·unbound_) (Fig. 5 *A* and *B*). The overall SetA_CTD_ structure consists of a four-α-helix bundle (FHB) with a long β-hairpin located in between helices αA and αB (Fig. 5*B* and *SI Appendix*, Fig. S7). The structure of SetA_CTD_·unbound revealed an additional helix (αE) at the C-terminal region and a 3_10_ helix between αC and αD (Fig. 5*B*). Notably, SetA_CTD_ shares the common features of an FHB fold and PI-binding ability with effectors/toxins containing a PI-binding domain (30–32) (*SI Appendix*, Fig. S7), rather than typical PI3P-binding domains, such as an FYVE (or an FYVE zinc-finger domain) or a Phox (PX) domain (33, 34).

The structures of SetA_CTD_·unbound and SetA_CTD_·PI3P reveal markedly different conformations in their binding sites for PI3P (Fig. 5 *A–D*). Upon PI3P binding, the surface becomes positively charged, driven by shifts in the αA-βB loop and αC-αD region. The αA-βB loop moves outward (~ 6.6 Å) from the binding cleft, exposing the hydrophobic side chains of Leu549 and Leu550 to open the binding site entrance (Fig. 5 *D* and *E*). The αA-βB loop changes are coupled with rearrangements of the αC-αD region, collapsing the 3_10_ helix to generate an unwound loop with an ~ 11.3 Å inward shift of Asn610^Cα^ and facilitating a major rotation of the bulky side chains of His606, Phe608, and Phe609 (Fig. 5 *D* and *E*). These rearrangements generate a positive patch involving His606 and Arg612 that accommodates the negatively charged PI3P head group. In addition, it is noteworthy that the upward reorientation of Leu549, Leu550, Phe608, and Phe609 aligns with the D1 phosphate of PI3P, which was linked to the aliphatic lipid acyl chains of the membrane (Fig. 5*D*). This suggests that the hydrophobic consecutive residues on αA-βB and αC-αD may insert into the membrane bilayer during the binding of membrane-embedded PI3P to SetA_CTD_–similar to PI4P recognition by the PI4P-binding domain of SidC (*SI Appendix*, Fig. S7) (31).

The structure of SetA_CTD_·PI3P also highlighted structural features for PI selectivity. For instance, SetA_CTD_ specifically recognizes the PI3P D3 phosphate via Asn567^N^, Gly568^N^, His606^Nε2^, Arg612^Nε,Nη1^, and Tyr620^Oη^ (Fig. 5*E*). Among them, Arg612 mainly contributes to a positive lobe near the open end of the central pocket to accommodate the D3 phosphate (Fig. 5*E*). Lipid-binding assays using three double mutants (K577Q/K578Q, K595Q/K598Q, and R605Q/K621Q) and one single mutant (R612Q) of SetA_CTD_ showed that only the R612Q single mutation abolished PI3P binding, while other mutants maintained or slightly reduced their capacity to bind PI3P, confirming that Arg612 is essential for PI3P recognition (Fig. 5*F* and *SI Appendix*, Fig. S6). The D1 phosphate of PI3P is stabilized through hydrogen bonds with Asn567^Nδ2^ and Asn610^Nδ2^ (Fig. 5*E*). Additional interactions between the 2′ hydroxyl group of the inositol ring and Asn567^N,Oδ1,Nδ2^ further orient the ligand within the binding pocket, positioning the 3′–6′ hydroxyl groups toward the inner pocket. Moreover, the αA-βB and αC-αD loops, particularly Gly548^N^, Leu550^O^, and Gln611^O^, form hydrogen bonds with the 5′ and 6′ hydroxyl groups of the inositol ring (Fig. 5*E*). The partial arrangement of these two loops, however, creates significant steric hindrance at the D4 phosphate position, explaining why PI4P and PI(4,5)P_2_ are strongly disfavored, providing a structural basis for the preference of SetA for PI3P. Together, these structural features not only provide PI selectivity but also ensure the proper positioning of the D1 phosphate, which is potentially connected to the lipid acyl chains of the LCV membrane.

### Spatial Localization and Structural Integration of SetA Supporting Rab1-Targeted Activity.

SetA predominantly anchors at PI3P-containing LCVs during the early phase of infection, where Rab1 is highly enriched. Given that SetA_NTD_ specifically recognizes and modifies Rab1 at its *O*-glucosylation motif, we reasoned that SetA perturbs the Rab1-dependent ER-to-Golgi trafficking machinery. As a small GTPase, Rab1 controls vesicle formation, movement, and fusion, making it a key target for SetA-mediated interference (35). This mechanistic link prompted us to further investigate the subcellular localization of SetA and its impact on organelle structure.

**Fig. 6.**
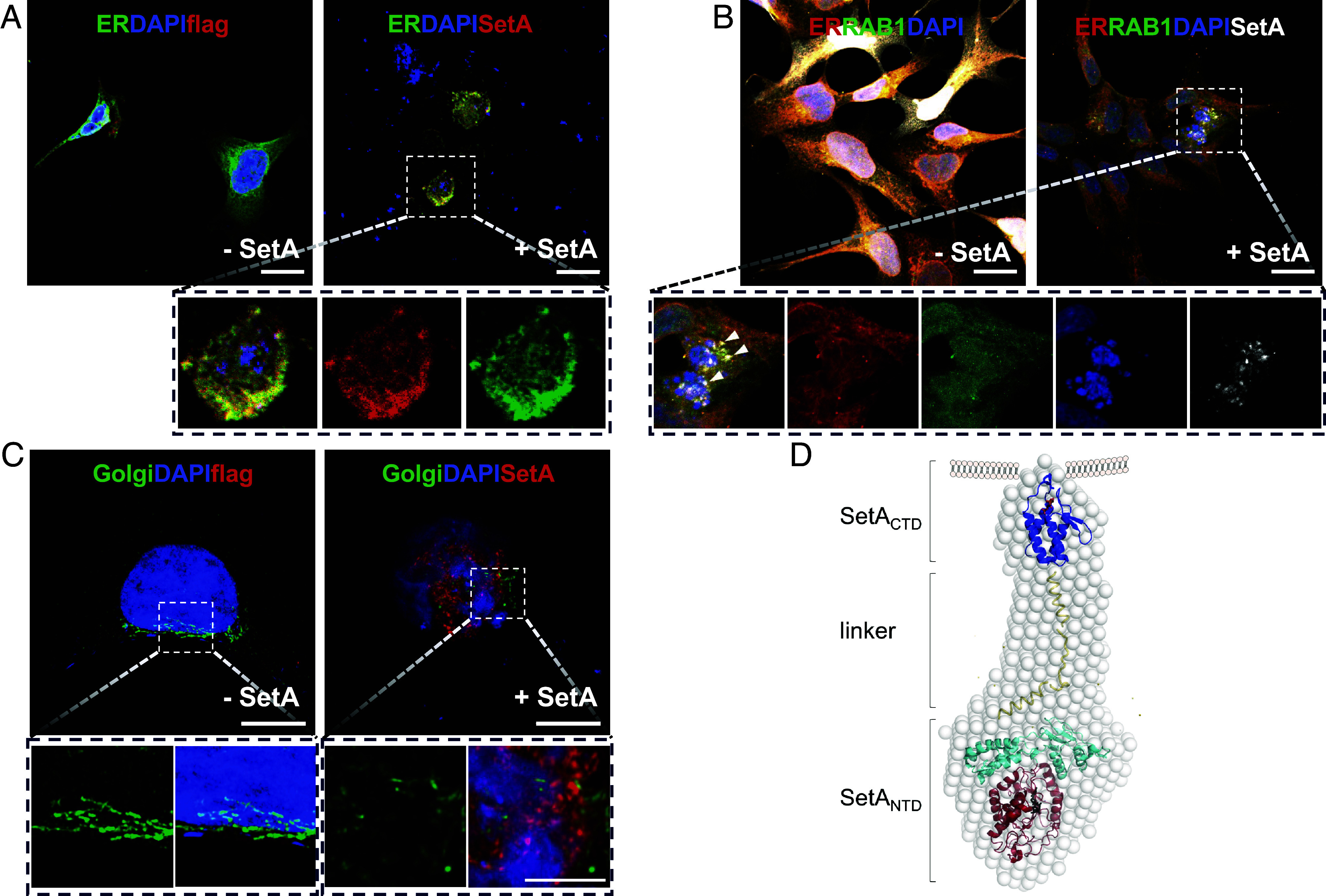
Cellular localization and the integrated model of SetA. (*A*) Confocal images of HEK293 cells transfected with empty vector (−SetA) or SetA (+SetA). Endoplasmic reticulum (ER) was labeled with calnexin (green), nuclei with DAPI (blue), and SetA/Flag with antibody staining (red). SetA shows extensive ER colocalization. (Scale bar, 20 µm.) (*B*) Triple staining of ER (red), Rab1 (green), and SetA (white) demonstrates spatial overlap of SetA with ER regions enriched in Rab1. For visualization, the SetA/Flag channel is displayed in white (single-channel pseudocolor) instead of red in this panel; the underlying data are identical to the red channel used elsewhere. Dashed boxes indicate areas shown at higher magnification. (Scale bar, 20 µm.) (*C*) Golgi apparatus labeled with GM130 (green) in cells ±SetA. SetA overexpression causes Golgi fragmentation and dispersion from the compact juxtanuclear ribbon; higher-magnification views highlight partial codistribution of SetA with reorganized Golgi membranes. (Scale bar, 10 µm.) (*A*–*C*) Dashed boxes indicate areas shown at higher magnification below, where individual channels are presented separately to highlight signal overlap. (*D*) Schematic integrative model illustrating the relative placement of structured SetA domains within the SAXS-derived molecular envelope. The N-terminal domain (SetA_NTD_; red and cyan) and the C-terminal domain (SetA_CTD_; blue) are manually positioned within the SAXS envelope (gray spheres) based on domain length, sequence connectivity, and overall envelope geometry. The intervening flexible linker region (yellow) is shown schematically.

Confocal imaging of HEK293 cells expressing SetA_full_ revealed strong colocalization with the ER marker calnexin (Fig. 6 *A* and *B*), and a partial overlap with the Golgi marker GM130, particularly in cells displaying disrupted Golgi morphology (Fig. 6*C*). Upon SetA overexpression, the Golgi apparatus lost its typical compact juxtanuclear structure and appeared fragmented or dispersed, while the ER exhibited altered topology, such as increased perinuclear compaction or reticulation. Considering that GM130 is a cis-Golgi matrix protein essential for maintaining Golgi ribbon integrity, such fragmentation reflects a disruption of cisternae connectivity and possibly a severe inhibition of vesicle trafficking rather than a nonspecific stress response. Higher magnification images revealed that SetA signal partially codistributes with the redistributed GM130 signal, indicating a direct spatial association of SetA with reorganized Golgi membranes (Fig. 6*C*). In cells exhibiting more pronounced Golgi disruption, SetA was found in punctate or aggregated structures near perinuclear regions, resembling the LCV-like compartments typical of early *Legionella* infection. These organelle disruptions likely underlie the cytotoxic phenotype induced by SetA overexpression (Fig. 1*A*). These observations suggest that SetA engages or intercepts Rab1 on PI3P-enriched early LCV membranes or LCV-associated ER-derived compartments, leading to Golgi fragmentation through interference with ER-to-Golgi vesicular trafficking. While PI3P is typically an endosomal marker, its transient presence on the early LCV allows SetA to spatially position itself to intercept Rab1 during the initial stages of vacuole remodeling.

To construct a comprehensive structural model, we incorporated the crystal structures of SetA_NTD_ and SetA_CTD_ into the SAXS-derived envelope of SetA_full∆13_, guided by domain shape, size, and sequential order (Fig. 6*D*). The ligand-bound structures of the SetA_NTD_ domain consisting of SetA_NTD·GTase_ and SetA_NTD·Cap_ were placed into the large, arrowhead-shaped region of the SAXS envelope, corresponding to their enzymatic core role. The C-terminal SetA_CTD_ domain, responsible for PI3P binding, fits well into the smaller globular region at the tail end. Its orientation was supported by the PI3P-bound structure, where a cluster of hydrophobic residues mediates membrane association, positioning SetA_CTD_ toward the membrane interface. The intervening segment (residues 475 to 528), predicted to be intrinsically disordered, likely serves as a flexible linker bridging the SetA_NTD_ GTase and membrane-localization modules (Fig. 6*D*). This spatial and structural organization integrates biochemical function with infection-stage localization, providing a coherent mechanistic framework for SetA-mediated host cell modulation during *Legionella* infection.

## Discussion

Considering the growing prevalence of Legionnaires’ disease, a molecular understanding of its infection process is crucial for identifying new therapeutic targets. Our study focuses on SetA, a *Legionella* glucosyltransferase implicated in the maturation of LCVs. To address the gaps in our knowledge of the molecular function of SetA, we employed structural, biochemical, and cellular approaches to uncover how SetA glucosylates its substrate Rab1 via its N-terminal domain (SetA_NTD_) and anchors to host membranes through its PI3P-specific C-terminal domain (SetA_CTD_).

The crystal structures of SetA_NTD_ in its unbound, substrate (UDP·Glc)-bound, and product (UDP)-bound states revealed a distinct GT-A fold and highlighted conformational transitions that prime the active site for interaction with the acceptor substrate Rab1. While the SetA_NTD·GTase_ domain exhibits a canonical type-A glycosyltransferase (GT-A) fold conserved among various bacterial glucosyltransferases, such as Lgt1-3 from *L. pneumophila* (36), ToxG from *Photorhabdus asymbiotica* (37), and Toxin A (TcdA) or Toxin B (TcdB) from *Clostridium difficile* (38, 39) (*SI Appendix*, Figs. S8 *A* and *B*), their substrate engagement strategies differ from that of SetA in terms of substrate specificity, with each recognizing distinct target proteins depending on their domain or subdomain configuration. Notably, structural analyses revealed that SetA_NTD_ comprises the GTase core domain (SetA_NTD·GTase_) and a unique core-associated subdomain (SetA_NTD·Cap_), the latter positioned atop the GTase core and acting as a protective shield (Fig. 2*A*). This arrangement suggests that Rab1 accesses SetA_NTD·GTase_ from below, and the binding of the donor substrate UDP·Glc triggers an expansion of the GTase domain cavity, facilitating Rab1 entry (Fig. 3*H*). Indeed, the docking of the Rab-derived peptide into the expanded cavity of SetA_NTD_ (Fig. 4*D*) provided a clear illustration of the biophysical interactions between SetA and its recognition site on Rab1. These insights allowed us to generate a qualitative integrative model of full-length Rab1 protein bound to SetA_NTD_ (*SI Appendix*, Fig. S9). While purely predictive, this model offers a plausible structural rationale for the enhanced binding affinity of intact Rab1 protein relative to the Rab1 peptide (Fig. 4), potentially involving additional contacts beyond the Switch II loop, such as the a3 helix of Rab1. In contrast, other GTases, such as Lgt1, TcdA/B, and ToxG, likely recruit substrates differently owing to variations in the placement of their subdomains (*SI Appendix*, Fig. S8). This underscores the importance of the subdomain structure in substrate specificity despite the commonality of the GTase core domain, illustrating a physiologically plausible mode of substrate recognition and modification that appears unique among related GTases.

Our structures of SetA_CTD_ in both PI3P-bound and unbound forms (Fig. 5 *A*–*D*) reveal that the membrane-targeting mechanism displays a remarkable parallel to that of the PI4P-binding effector SidM/DrrA. Del Campo et al. established that SidM achieves high-avidity recruitment through a combination of head-group coordination and the insertion of a hydrophobic helical element into the membrane (40). In SidM, this Membrane Insertion Motif (MIM) is defined by a cluster of leucines (L610, L614, L615, and L617) that reorient to penetrate the hydrocarbon core of the lipid bilayer, thereby compensating for the relatively low affinity of the soluble head group alone. Structural comparison reveals that SetA utilizes a functionally analogous hydrophobic cluster (Leu549, Leu550, Phe608, and Phe609), which, upon PI3P binding, is positioned to face the lipid interface in a manner similar to the SidM MIM (*SI Appendix*, Fig. S10). Although the two proteins utilize very different scaffolds, they appear to have reached a convergent biophysical solution for membrane anchoring (*SI Appendix*, Fig. S10).

The structures of SetA_CTD_ reveal a distinctive four-helix bundle (FHB) architecture that supports high-affinity association with PI3P-enriched membranes. Structural and mutational analyses identify Arg612 as a key residue coordinating the D3 phosphate, thereby conferring specificity for PI3P. Consistent with this, protein–lipid overlay assays demonstrate a strong biochemical preference for PI3P, although a much weaker interaction with PI(3,5)P_2_ was also detected. Structurally, although the PI3P-binding pocket appears sterically constrained, the D5 position of the inositol ring is oriented toward a relatively more solvent-accessible region compared to the D4 position. Together with the conformational flexibility of the αA–βB and αC–αD loops, this arrangement suggests that limited local rearrangements could permit accommodation of the additional phosphate present in PI(3,5)P_2_. Given that the *Legionella* effector SidP is responsible for the removal of both PI3P and PI(3,5)P_2_ from the early LCV (7), the ability of SetA to recognize both lipids may facilitate sustained membrane association prior to vacuole maturation.

Our cell imaging analyses revealed that SetA overexpression causes pronounced ER compaction and Golgi fragmentation (Fig. 6 *A*–*C*). Together with the structural basis of Rab1 and PI3P recognition, these observations indicate that SetA perturbs Rab1-dependent ER–Golgi trafficking. We propose that SetA-mediated Rab1 interference disrupts early secretory trafficking by impairing vesicle formation or tethering at the ER–Golgi interface. This disruption likely blocks protein and lipid flow to the Golgi and prevents GM130 from maintaining cis-Golgi organization, ultimately leading to disintegration of the Golgi ribbon. Thus, SetA-induced remodeling reflects not classical ER stress but a deliberate structural reprogramming of the secretory pathway by SetA as part of *Legionella*’s infection strategy. Together, these findings further illustrate the sophisticated mechanism of pathogen–host interactions, demonstrating how a single bacterial protein such as SetA can actively restructure host organelles by integrating precise membrane localization with targeted enzymatic activity. Perturbation of these compartments may compromise essential biosynthetic and secretory pathways, potentially resulting in growth arrest or stress-induced cell death.

The recognition of PI3P provides a critical temporal window for SetA function during the early stages of infection. A previous study using live-cell imaging showed that PI3P is acquired rapidly within a minute after *Legionella* uptake and is lost within 2 h (26), coinciding with the transition of the LCV from an endocytic to a secretory-like compartment. This finding suggests that SetA acts as an early upstream modulator that primes the nascent LCV for subsequent remodeling. Anchored to the early PI3P-rich membrane, SetA directly interacts with Rab1-GDP and glucosylates it (Fig. 4). This modification may facilitate the dissociation of Rab1-GDP from its GDI complex, thereby enabling its spatial repositioning into the PI3P-enriched early LCV environment.

This SetA-mediated priming and positioning of Rab1 precedes the Rab1 activation phase mediated by other effectors. As the LCV matures, PI3P is cleared and PI4P gradually accumulates on the membrane over the subsequent 8 h (26). At this stage, PI4P-binding effectors including SidM and LidA are recruited to the LCV, where SidM activates Rab1 to its GTP-bound state at the LCV, after which LidA and other tethering factors engage Rab1 to facilitate the recruitment and fusion of ER-derived vesicles, driving vacuole maturation (41–47). While SidM is translocated within ~30 min of infection (26), the ability of SetA to target even earlier PI3P-enriched membranes allows it to establish an initial pool of membrane-associated Rab1 before SidM-dependent activation occurs. Furthermore, Rab1 glucosylation does not inhibit the GEF activity of SidM (21), suggesting a hierarchical ordering of modifications in which SetA-mediated Rab1 modification occurs early during LCV formation.

Together, these findings clarify the sequential but partially overlapping mechanisms underlying early LCV formation. First, SetA utilizes its structural feature to anchor onto early PI3P/PI(3,5)P_2_-enriched membranes. Subsequently, SetA glucosylates GDP-bound Rab1 to promote GDI displacement, thereby ensuring that a sufficient pool of Rab1 is spatially positioned for subsequent functional activation by the PI4P-dependent effector machinery.

In conclusion, our comprehensive structural, biophysical, biochemical, cellular, and in silico docking analyses of SetA provide significant insights into its dual role in substrate glucosylation and PI3P recognition during early LCV formation. These findings enhance our understanding of *Legionella* pathogenesis by detailing the molecular mechanisms through which translocated effectors coordinate enzymatic activity with precise spatial positioning to establish a replicative niche.

## Methods

### Expression and Purification of Recombinant Proteins.

Recombinant SetA constructs were cloned into pET-21a(+), expressed in *E. coli* strains Rosetta 2(DE3) or Rosetta 2(DE3)pLysS, and induced at low temperature for soluble production. Proteins were purified using Ni^2+^-affinity chromatography followed by size-exclusion chromatography, yielding >95% purity. Concentrated native and SeMet-labeled proteins were prepared for crystallization under identical purification conditions and are described in more detail in *SI Appendix*, *Methods*.

### SAXS.

SetA_full∆13_ proteins were concentrated to 11.3 mg ml^-1^ and subjected to SAXS measurements at beamline BL-4C (Pohang Light Source) with reference and dilution series profiles collected to assess radiation damage and concentration effects. The SAXS data collection and analysis statistics are presented in *SI Appendix*, Table S1. SAXS data acquisition and analysis procedures are described in more detail in *SI Appendix*, *Methods*.

### Crystallization, Data Collection, and Structure Determination.

Crystallization of SetA_NTD_ and SetA_CTD_ was carried out by sitting-drop vapor diffusion under multiple optimized conditions for unbound, substrate-bound, product-bound, and PI3P-bound states, with analogous setups used for SeMet-labeled proteins. Diffraction data were collected at Pohang Light Source and SPring-8, processed with HKL2000 or XDS, and structures were solved by SAD or molecular replacement followed by iterative model building and refinement. *SI Appendix*, Tables S2 and S3 present the phasing and model refinement statistics, respectively. Crystallization, data collection, phasing, and refinement procedures are described in more detail in *SI Appendix*, *Methods*.

### SEC-MALS.

Size-exclusion chromatography with multiangle light scattering (SEC-MALS) experiments for SetA_full_, SetA_NTD(14–474)_, SetA_linker·CTD_, and SetA_CTD_ were performed using an FPLC system. Experimental setup and data-processing procedures are described in more detail in *SI Appendix*, *Methods*.

### In Vitro Glucosylation of Synthetic Peptides and Mass Spectrometry.

Synthetic peptides were analyzed by nano-LC–MS/MS using an Ultimate 3000 RSLCnano system coupled to a Q Exactive HF-X Orbitrap, with separation on C18 columns under a 90-min gradient. Data-dependent acquisition (top-20) was performed with high-resolution MS and HCD MS/MS to identify glucosylated products. Full experimental details are provided in *SI Appendix*, *Methods*.

### UDP-Glc Hydrolase Activity Assay.

The relative hydrolysis activities of SetA_full_ wild-type (WT) or mutants (W36A, R121A, Y132A, D134A/D136A, D136A, and K210A) were studied by measuring the release of free UDP using an ADP Quest Assay kit (DiscoverRx) according to the manufacturer’s protocol. Full experimental details are provided in *SI Appendix*, *Methods*.

### SPR Analysis.

SetA_NTD_ binding kinetics and affinities for Rab1 proteins and Rab1-derived peptides were quantified by SPR using Reichert SR7500 and Biacore T200 instruments. Association/dissociation rates were recorded under defined running buffers, and sensorgrams were fitted using Scrubber2 or Biacore evaluation software. Full SPR experimental conditions and analysis procedures are described in more detail in *SI Appendix*, *Methods*.

### MST.

The binding affinity between SetA_NTD_ and UDP-Glc was determined using a Monolith NT.115 instrument (NanoTemper) at 22 °C and measured at 30% LED and 40% MST power with a 30 s laser on time and 5 s laser off time. Full MST experimental conditions and analysis procedures are described in more detail in *SI Appendix*, *Methods*.

### Protein–Lipid Overlay Assay.

To test the direct binding of SetA_CTD_ to lipids, experiments were carried out with the purified SetA_CTD_ WT or double mutants (K577Q/K578Q, K595Q/K598Q, and R605Q/K621Q) using PIP Strips^TM^ or a Custom PI3P. Full experimental conditions are described in more detail in *SI Appendix*, *Methods*.

### Cell proliferation.

HEK293 cell growth was assessed after transfection with control or SetA-expressing plasmids, followed by daily fixation, permeabilization, DAPI staining, and quantification using a Cytation 3 imaging system. Full experimental conditions are described in more detail in *SI Appendix*, *Methods*.

### Immunofluorescence.

Immunofluorescence microscopy was performed on fixed, permeabilized cells stained with antibodies against Flag, GM130, Calnexin, and Rab1, followed by fluorescent secondary antibodies and DAPI. Full experimental details are provided in *SI Appendix*, *Methods*.

## Supplementary Material

Appendix 01 (PDF)

## Data Availability

The coordinates and structure factors were deposited in the Protein Data Bank under accession codes 8X4J (48), 8X4K (49), 8X4M (50), and 8X4N (51) for SetA_NTD_, SetA_NTD_·UDP·Glc, SetA_NTD_·UDP, and SetA_CTD_, respectively. Study data are included in the article and/or *SI Appendix*.
