# Supplementary material for: Structural insights into SetA-mediated Rab1 glucosylation and PI3P-guided localization during early Legionella infection
Source: Proc Natl Acad Sci U S A. 2026 Mar 27;123(13):e2535016123. doi: 10.1073/pnas.2535016123 (PMC13037854; doi:10.1073/pnas.2535016123)
Supplement: Supplementary file 1 — Appendix 01 (PDF) [file pnas.2535016123.sapp.pdf]

PNAS

Supporting Information for

Structural insights into SetA-mediated Rab1 glucosylation and PI3P-guided localization during early *Legionella* infection

Ha Na Im[1,2,8], Yeon Lee[1,8], Yunju Song[1,3,8], Hyunggu Hahn[1], Hyerry Jeon[1], Donghyuk Shin[4], Sangho Lee[5], Kyung-Hee Kim[1,6], Kyung-Tae Kim[1], Se Won Suh[2], Dong Man Jang[1,7*] & Hyoun Sook Kim[1*]

Dong Man Jang[1,7*] & Hyoun Sook Kim[1*]

Email: djang@crystal.harvard.edu (DMJ) & hskim@ncc.re.kr (HSK)

This PDF file includes:

Tables S1 to S3

Figures S1 to S10

Methods

SI References

Table S1. SAXS data collection and analysis statistics of $SetA_{full\Delta13}$

Data-collection parameters	
Synchrotron beamlines	PAL-4C
Beam geometry	Capillary
Wavelength (Å)	1.24
Exposure time (s)	10
Concentration range (mg/ml)	1.42 – 11.32
Structural parameters	
I(0) (cm^{-1}) [from Guinier]	15.35
R_g (Å) [from Guinier]	47.01
I(0) (cm^{-1}) [from P(r)]	15.36
R_g (Å) [from P(r)]	47.43
D_{max} (Å)	176
Porod volume estimate ($Å^3$)	131898
Software employed	
Primary data reduction	*RAW*
Dara processing	*PRIMUS*
Ab initio analysis	*DAMMIF*
Validation and averaging	*DAMAVER*
Three-dimensional representations	*PyMOL*

Table S2. Data collection and refinement statistics of $\mathrm{SetA_{NTD}}$

A. Data collection				
Data set	$\mathrm{SetA_{NTD}}$-peak	$\mathrm{SetA_{NTD}}$·unbound	$\mathrm{SetA_{NTD}}$·UDP·Glc	$\mathrm{SetA_{NTD}}$·UDP
Beam source	PLS BL-5C	PLS BL-5C	SPring-8 BL44XU	PLS BL-11C
Space group	$P3_221$	$P3_221$	*P*212121	*P*212121
a, b, c (Å)	140.3, 140.3, 60.1	139.4, 139.4, 59.9	42.1, 63.3, 185.0	42.8, 62.8, 176.7
α, β, γ (°)		90.0, 90.0, 120.0	90.0, 90.0, 90.0	90.0, 90.0, 90.0
X-ray wavelength (Å)	0.9795	0.9796	0.9999	0.9794
Resolution range (Å)	50.0 – 2.85 (2.90 – 2.85)[a]	42.54 – 2.54 (2.63 – 2.54)[a]	44.2 – 1.90 (2.0 – 1.90)[a]	29.6 – 1.80 (1.9 – 1.80)[a]
No. total reflections	327,509	237,383	298,248	214,489
No. unique reflections	15,693	42,822	75,186	44,091
Completeness (%)	97.6 (94.2)[a,b]	99.9 (99.3)[a]	99.9 (100.0)[a]	98.6 (93.1)[a]
$\langle I \rangle / \langle \sigma_I \rangle$	62.3 (4.7)[a,b]	48.1 (3.7)[b]	11.7 (1.2)[b]	15.5 (1.48)[b]
R_{sym}[c] (%)	9.3 (82.9)[a,b]	10.0 (91.2)[a]	5.9 (94.7)[a]	13.2 (89.1)[a]
R_{pim}[d] (%)	2.0 (18.7)[a,b]	3.3 (28.5)[a]	7.0 (111.4)[a]	6.9 (46.3)[a]
B. Model refinement				
PDB Entry		8X4J	8X4K	8X4M
Resolution range (Å)		42.54 – 2.54	44.2 – 1.90	29.6 – 1.80
R_{work}/R_{free}[e] (%)		19.3 / 24.7	17.5 / 21.1	20.1 / 23.8
No. of non-hydrogen atoms / average *B*-factor ($Å^2$)				
Protein		3,608 / 71.97	3,668 / 45.05	3,658 / 29.06
Water oxygen		54 / 62.39	196 / 49.66	289 / 36.6
Ligand		1 / 81.64	18 / 63.56	23 / 36.6
R.m.s. deviations from ideal geometry				
Bond lengths (Å) / angles (°)		0.008 / 1.30	0.009 / 1.46	0.006 / 1.09
Ramachandran (%)[f]				
Favored / Outliers		97.5 / 0.00	99.1 / 0.00	98.4 / 0.00
Poor rotamers (%)[f]		1.51	0.50	0.50

[a]Values in parentheses refer to the highest resolution shell.
[b]Friedel pairs were treated as separate observations.
[c]$R_{merge} = \Sigma_h\Sigma_i \mid I(h)_i - \langle I(h)\rangle \mid / \Sigma_h\Sigma_i I(h)_i$, where $I(h)$ is the intensity of reflection h, Σ_h is the sum of all reflections, and Σ_i is the sum of i measurements of reflection h.
[d]$R_{pim} = \sum_{hkl} (1 / (N(hkl) - 1))^{1/2} \sum_i \mid I_i(hkl) - \langle I(hkl)\rangle \mid / \sum_{hkl}\sum_i I_i(hkl)$. The precision of the merging R-factor describes the precision of the average intensity.
[e]$R_{work} = \Sigma \mid |F_{obs}| - |F_{calc}| \mid / \Sigma |F_{obs}|$, where R_{free} was calculated for a randomly chosen 5% of the reflections that were not used for structural refinement, and R_{work} was calculated for the remaining reflections.
[f]Values obtained using MolProbity.

Table S3. Data collection and refinement statistics of SetA_{CTD}

A. Data collection		
Data set	$\text{SetA}_{\text{CTD·3KA}}$-peak	SetA_{CTD} ·I13P
Beam source	PLS BL-5C	PLS BL-7A
Space group	$I4_1$	*C*21
a, b, c (Å)	80.9, 80.9, 195.8	113.2, 37.0, 72.4
α, β, γ (°)	90.0, 90.0, 90.0	90.0, 111.6, 90.0
X-ray wavelength (Å)	0.9795	0.9793
Resolution range (Å)	37.19 – 2.39 (2.49 – 2.39)[a]	29.51 – 2.46 (2.55 – 2.46)[a]
No. total reflections	495,932	39,626
No. unique reflections	24,666	10,267
Completeness (%)	99.0 (98.0)[a,b]	99.1 (95.1)[a]
$<I> / <\sigma_I>$[b]	54.1 (6.1)[a,b]	21.5 (3.2)[a]
R_{merge}[c] (%)	13.1 (78.5)[a,b]	11.6 (76.2)[a]
R_{pim}[d] (%)	3.1 (17.9)[a,b]	6.7 (43.7)[a]
B. Model refinement		
PDB ID code		8X4N
Resolution range (Å)		29.51 – 2.46
R_{work} / R_{free}[e] (%)		21.9 / 26.6
No. of non-hydrogen atoms / average *B*-factor ($Å^2$)		
Protein		1727 / 44.2
Water oxygen		94 / 38.0
Ligand		26 / 20.4
Bond lengths (Å) / angles (°)		0.006 / 1.08
Ramachandran (%)[f]		
Favored / Outliers		98.6 / 0.00
Poor rotamers (%)[f]		0.00

[a]Values in parentheses refer to the highest resolution shell.
[b]Friedel pairs were treated as separate observations.
[c]$R_{\text{merge}} = \Sigma_h\Sigma_i \mid I(h)_i - <I(h)> \mid / \Sigma_h\Sigma_i\, I(h)_i$, where $I(h)$ is the intensity of reflection h, Σ_h is the sum of all reflections, and Σ_i is the sum of i measurements of reflection h.
[d]$R_{\text{pim}} = \sum_{\text{hkl}} (1 / [N(\text{hkl}) - 1])^{1/2} \sum_i \mid I_i(\text{hkl}) - <I(\text{hkl})> \mid / \sum_{\text{hkl}}\sum_i I_i(\text{hkl})$. The precision of the merging R-factor describes the precision of the average intensity.
[e]$R_{\text{work}} = \Sigma \mid |F_{\text{obs}}| - |F_{\text{calc}}| \mid / \Sigma |F_{\text{obs}}|$, where R_{free} was calculated for a randomly chosen 5% of the reflections that were not used for structural refinement, and R_{work} was calculated for the remaining reflections.
[f]Values obtained using MolProbity.

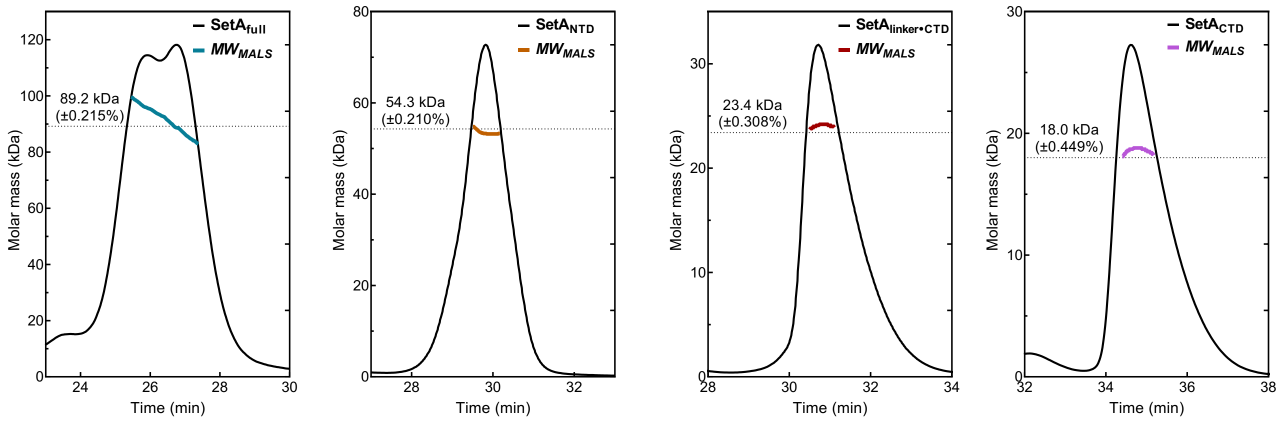

Figure S1. SEC-MALS analysis of the SetA constructs. Molar masses are plotted as a function of elution time for full-length SetA (89.2 kDa), SetANTD (54.3 kDa), SetAlinker·CTD (23.4 kDa), and SetACTD (18.0 kDa).

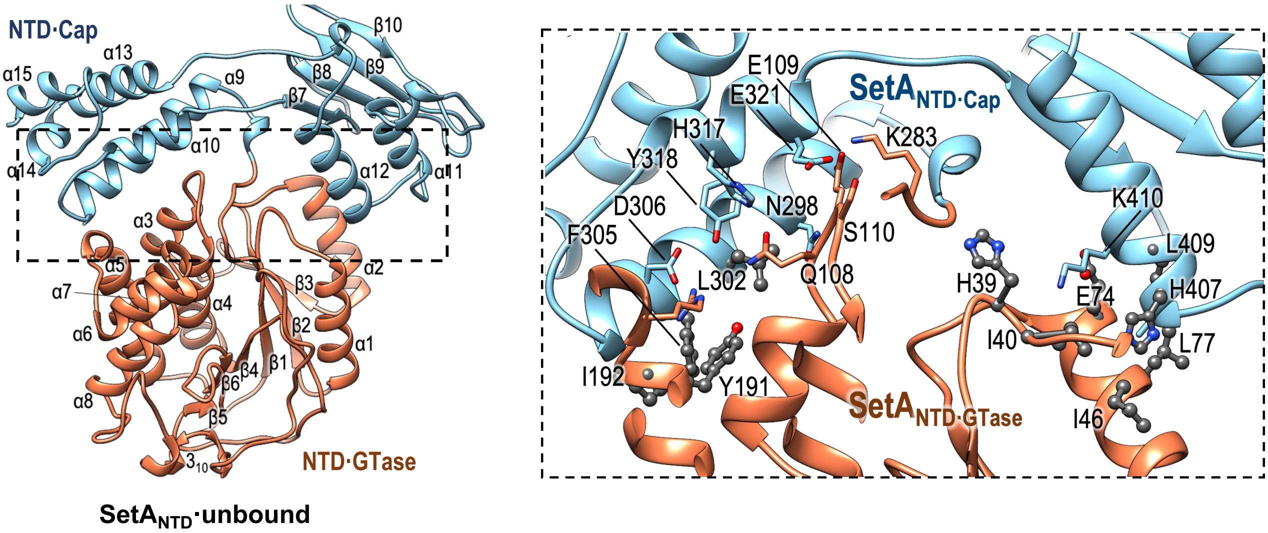

Figure S2. Overall structure of SetA$_{NTD·unbound}$. It shows the GTase (orange) and Cap (cyan) sub-domains. Secondary structures are displayed as cartoon representations. Close-up view of the interface between the GTase and Cap sub-domains, highlighting key residues involved in hydrophobic and hydrogen-bond interactions.

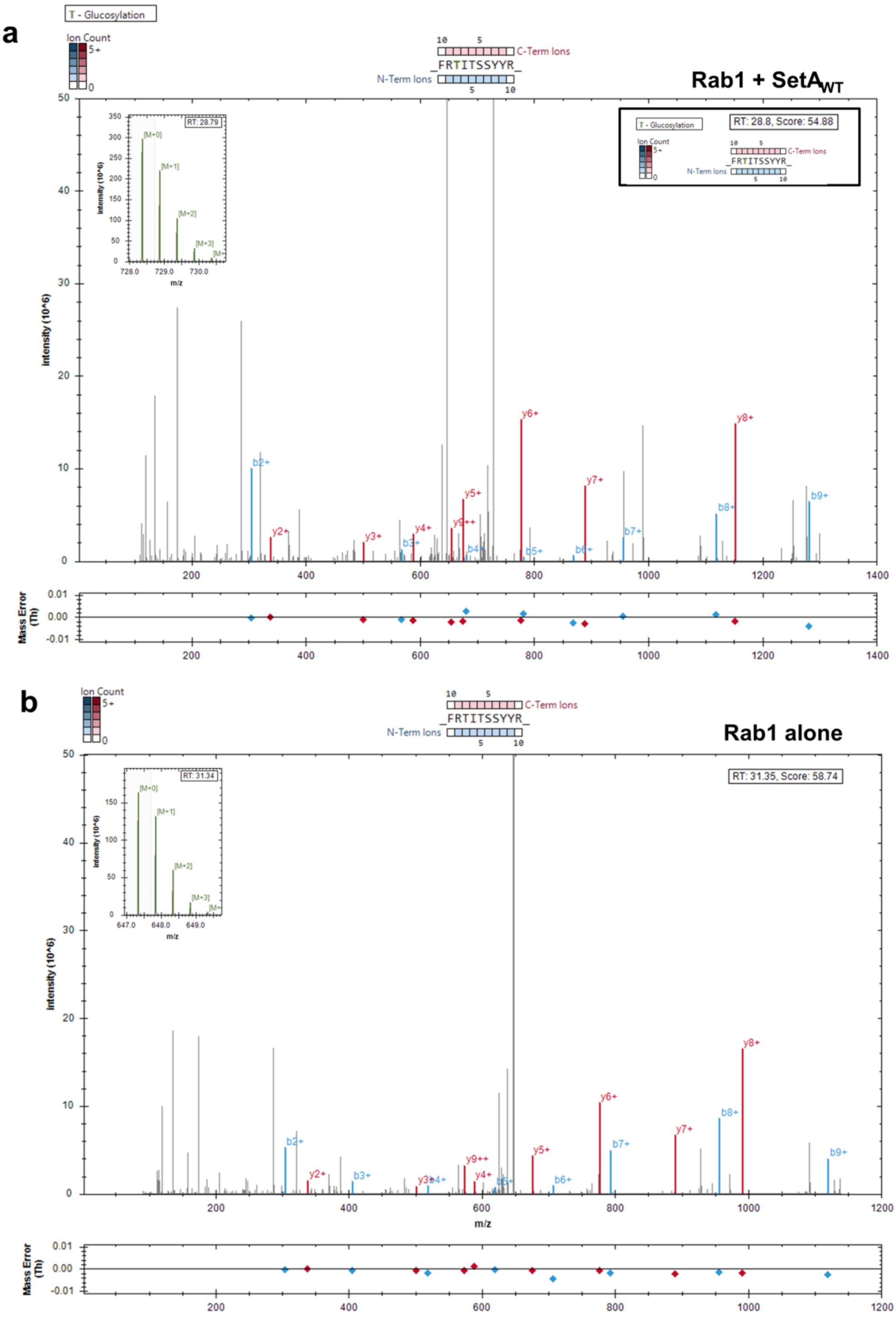

Figure S3. Identification of a SetA glucosylation site in Rab1 proteins. a,b Sequencing of the glucosylated peptide from Rab1 after incubation with SetA in the presence of UDP-Glc at 25°C for 12 h (**a**) or Rab1 alone (**b**) using LC-nanospray ionization tandem mass spectrometry (nanoLC-NSI/MS/MS). These results confirm that Rab1 is glucosylated at the Thr75 residue by SetA. The individual *b*- and *y*-ions are labeled in blue and red, respectively. The b3 and b4 ions unambiguously confirm glucosylation on Thr75. The MS/MS spectra on the MS2 analysis spectrum are shown in the top-left insets. The mass error plot below each main spectrum represents the deviation of identified b-ions (blue) and y-ions (red) from their expected masses. The y-axis indicates the mass error, and the x-axis corresponds to the m/z values of the ions. All errors remained within acceptable limits, confirming accurate mass measurements.

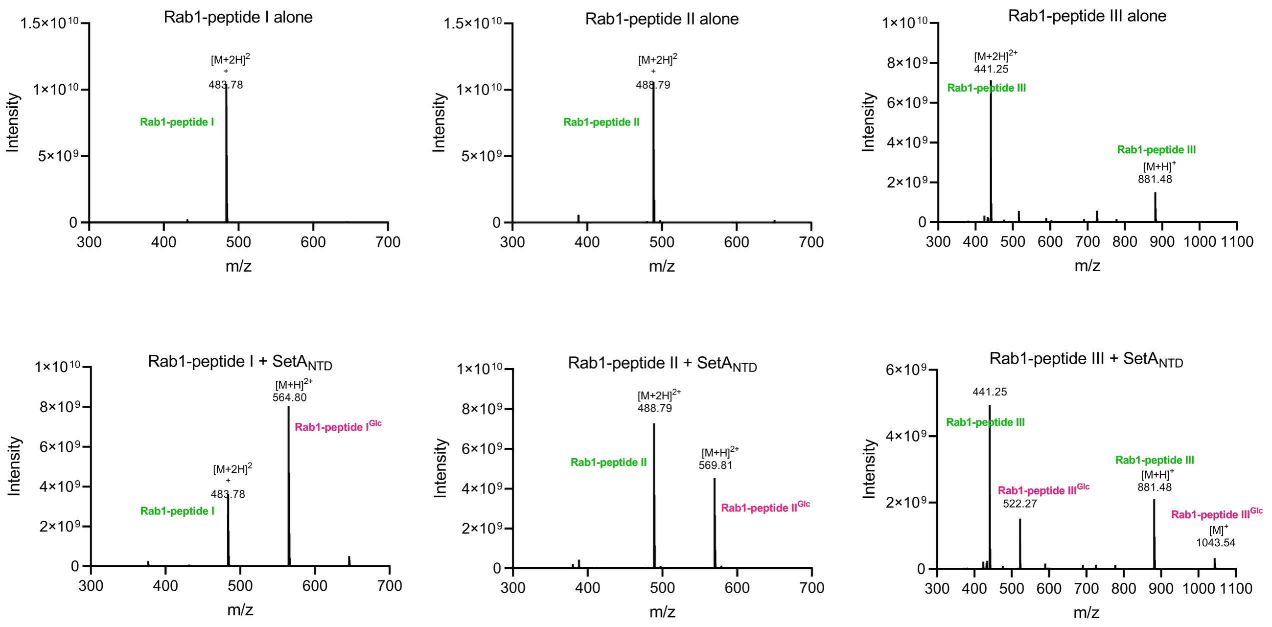

Figure S4. Mass spectrometry analysis of Rab1 peptide glucosylation by SetA. Mass spectrometry data showing the glucosylation of three different Rab1 peptides (Rab1 peptides I–III) by SetA. Unmodified peptides are shown in blue. Glucosylated forms (+162 Da) are marked in pink.

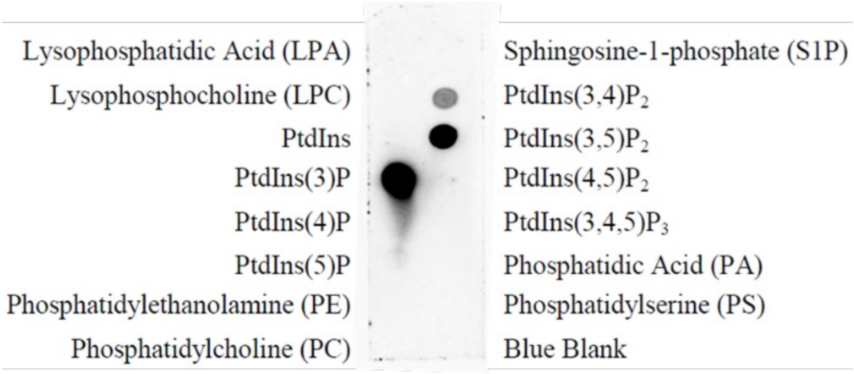

Full-length SetA

Figure S5. Evaluation of the lipid binding specificity of SetA$_{CTD}$ using protein-lipid overlay assays. Lipid blot showing the binding specificity of SetA$_{CTD}$ for various phospholipids. Different lipids, including phosphatidylinositol phosphates (PtdIns and PtdIns derivatives), phosphatidylethanolamine (PE), phosphatidylcholine (PC), phosphatidylserine (PS), lysophosphatidic acid (LPA), and sphingosine-1-phosphate (S1P), are spotted on the membrane.

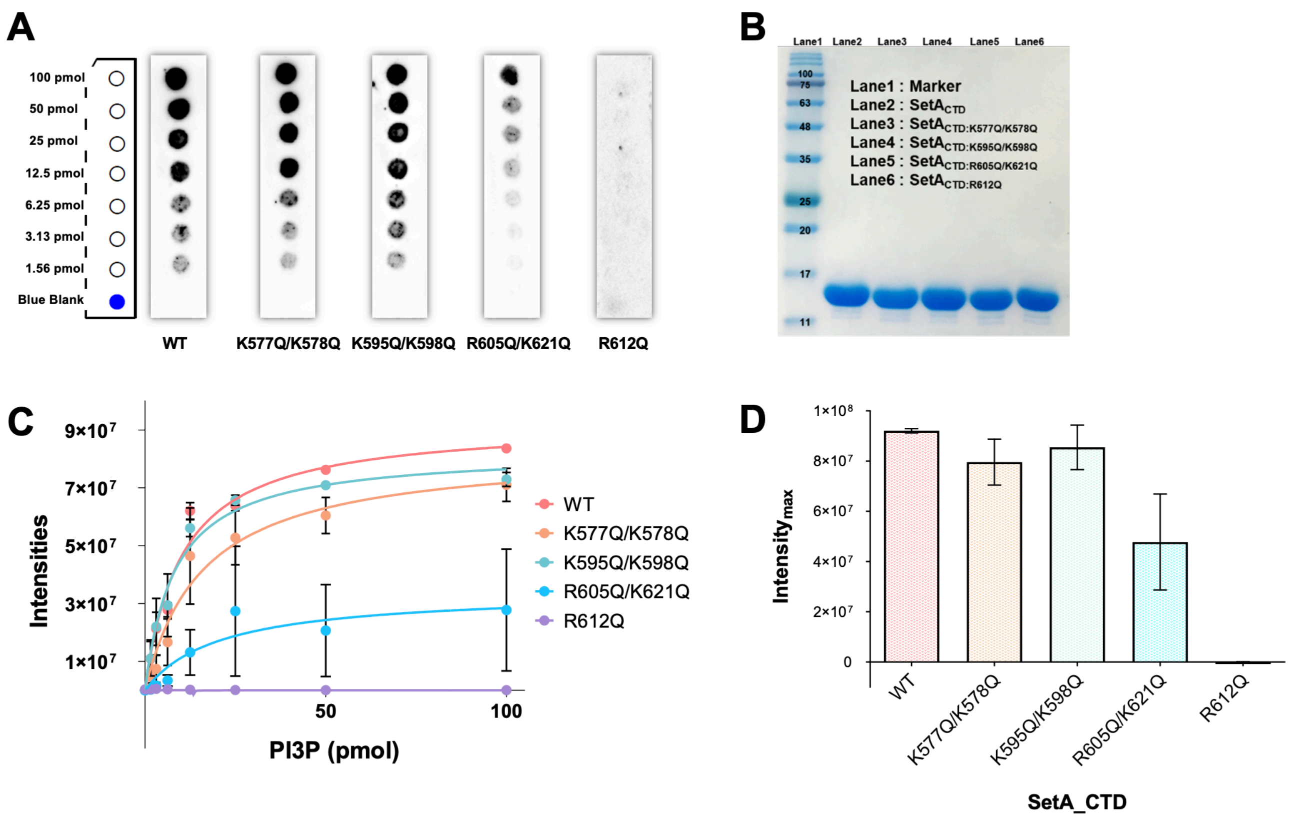

Figure S6. Mutational analysis of SetA$_{CTD}$ phosphoinositide binding. a Protein-lipid overlay assay showing the binding of SetA$_{CTD}$ and its mutants to phosphatidylinositol 3-phosphate (PI3P) at varying concentrations (1.56 to 100 pmol). Wild-type SetA$_{CTD}$ shows strong binding across all concentrations, while different mutations (K577Q/K578Q, K595Q/K598Q, R605Q/K621Q, R612Q) resulted in varying degrees of reduced binding, with R612Q showing the greatest reduction in PI3P binding. **b** SDS-PAGE analysis of purified SetA$_{CTD}$ proteins used in the protein-lipid overlay assay, confirming equal loading and purity of WT and mutant proteins. **c,d** Quantitative analysis of lipid binding intensities. Binding was normalized to wild-type SetA$_{CTD}$ (WT) and expressed as a relative index. Error bars represent the mean ± s.d. from three independent experiments.

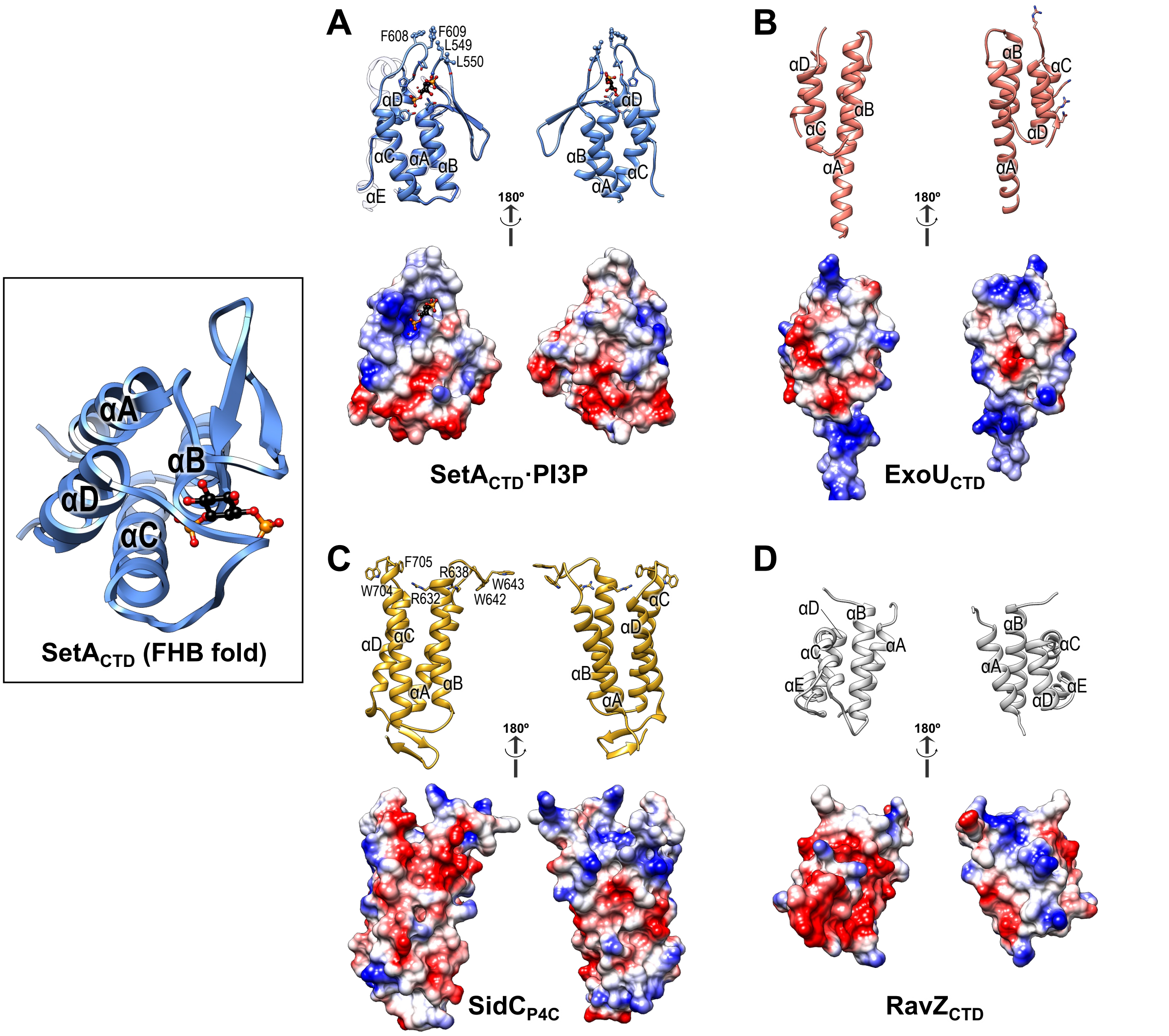

Figure S7. Structural comparison of SetA$_{CTD}$ with other effectors/toxins containing a phosphoinositide-binding domain. The structures of SetA$_{CTD}$ (**a**), ExoU$_{CTD}$ (a phospholipase from *Pseudomonas aeruginosa*, PDB: 3TU3) (**b**), SidC$_{P4C}$ (a phosphoinositide-binding domain of SidC from *Legionella pneumophila*, PDB: 4ZUZ) (**c**), and RavZ$_{CTD}$ (an effector protein from *L. pneumophila*, PDB: 5MS2) (**d**) are shown in both cartoon representations (top) and electrostatic surface representations (bottom), highlighting structural similarities and differences. The left inset highlights the four-α-helix bundle (FHB) of SetA$_{CTD}$ in cartoon representation with the bound PI3P moiety in stick representation.

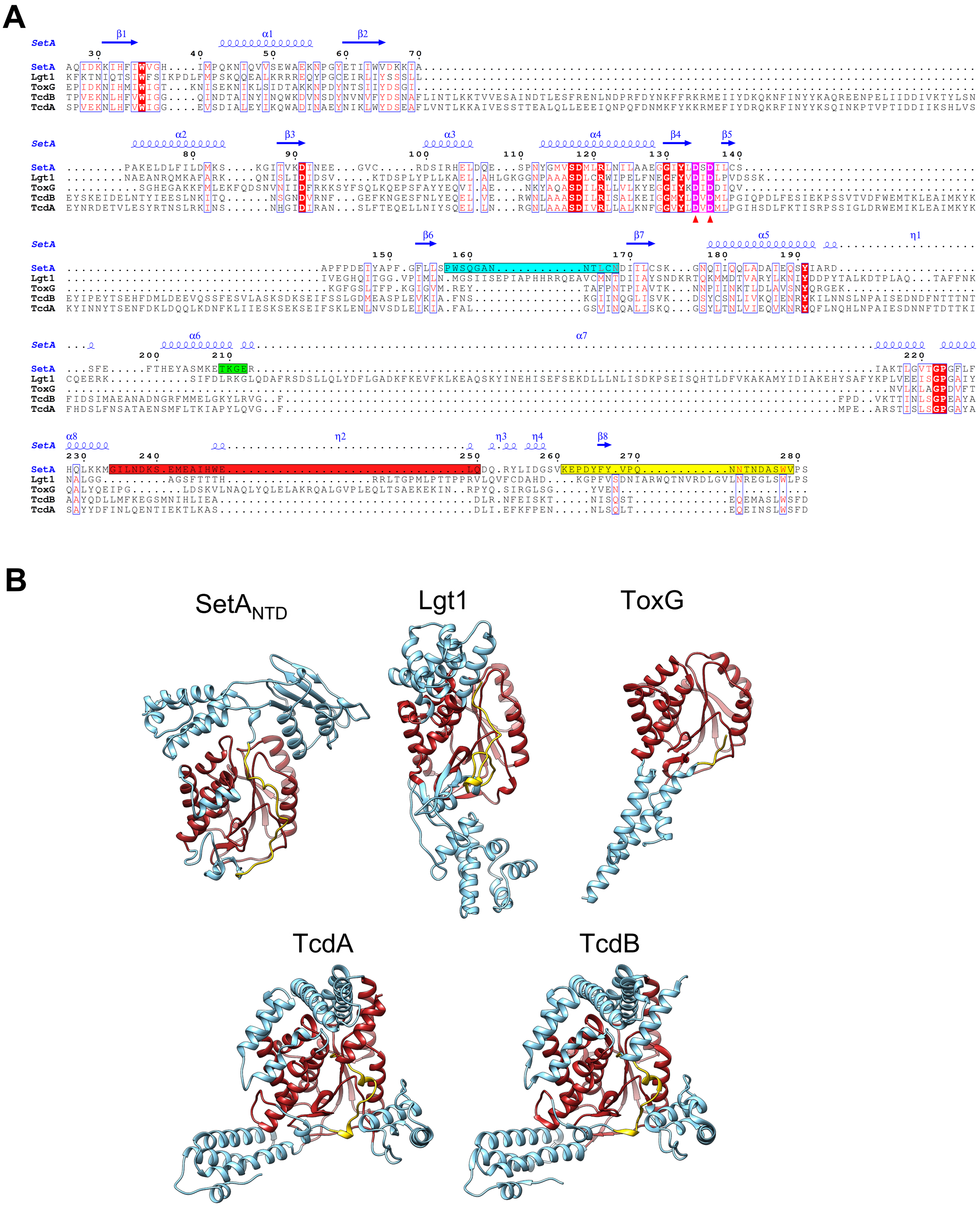

Figure S8. Sequence alignment and structural comparison of SetA$_{NTD}$ with related glucosyltransferases. **a** Sequence alignment of SetA$_{NTD}$ with other glucosyltransferases, including Lgt1–3 from *Legionella pneumophila,* ToxG from *Photorhabdus asymbiotica*, and Toxin A (TcdA) or Toxin B (TcdB) from *Clostridium difficile*. Conserved residues are highlighted in red, and secondary structure elements (α-helices and β-strands) are labeled according to the structure of SetA. Key catalytic residues are indicated by triangles. **b** Cartoon representations of SetA$_{NTD}$ and related glucosyltransferases, including Lgt1, ToxG, TcdA, and TcdB. The conserved core structural elements are highlighted in red, while variable regions are shown in blue. The structural similarities reflect the common mechanism of glucosyltransferase activity, while differences in the flexible regions likely account for substrate specificity across these enzymes. The structures used in this figure are from the following PDB entries: Lgt1 from *L. pneumophila* (PDB: 3JSZ), ToxG from *P. asymbiotica* (PDB: 4MIX) and Toxin A (TcdA) and Toxin B (TcdB) from *C. difficile* (PDB: 3SRZ and 2BVL, respectively).

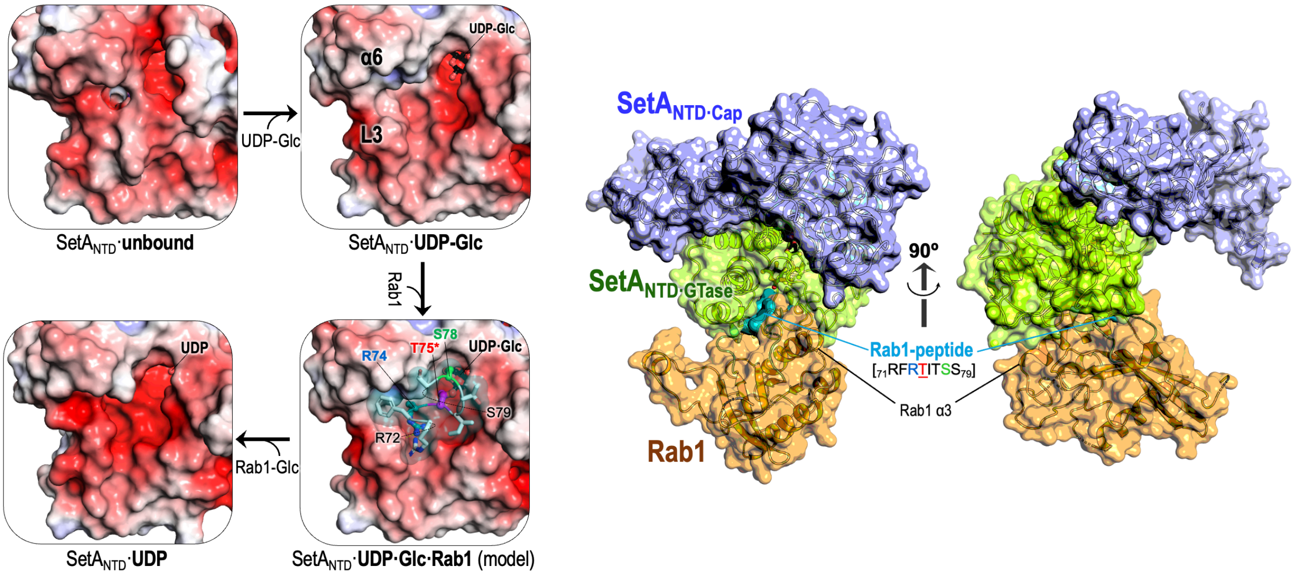

Figure S9. Comprehensive snapshots of SetA$_{NTD}$ complexes in multiple states.
(**Left**) Electrostatic surface representations of SetA$_{NTD}$ in different binding states investigated in this study. The docking model for the Rab1-peptide is shown as stick and cartoon representations. (**Right**) Cartoon and surface representation of the SetA$_{NTD}$-Rab1 complex, showing the GTase domain (green) and Cap domain (blue) of SetA$_{NTD}$ interacting with Rab1 (orange). The Rab1 peptide is shown as a cyan surface representation, highlighting its key contacts with SetA. The structural model suggests how SetA accommodates both UDP-Glc and Rab1, positioning Rab1 for glucosylation.

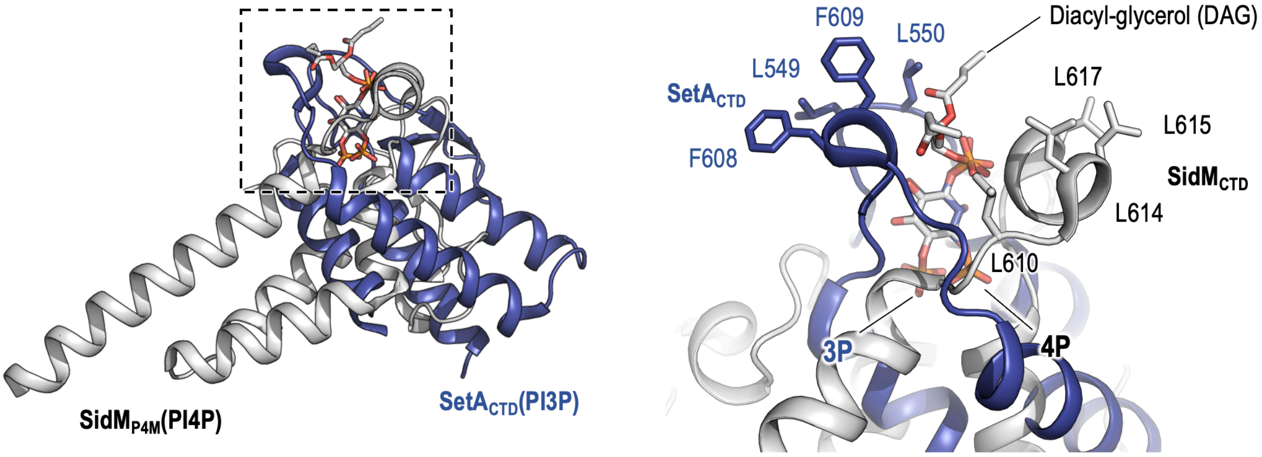

Figure S10. Structural comparison of SetA CTD and SidM.

Superposition of the SetA$_{CTD}$·PI3P structure with the SidM·PI4P structure (PDB: 4MXP) based on the phosphoinositide head group. Hydrophobic residues in SidM (L610, L614, L615, L617) and SetA$_{CTD}$ (Leu549, Leu550, Phe608, Phe609) are highlighted.

Methods

Expression and purification of recombinant proteins

The *lpg1978* gene encoding SetA was PCR-amplified from the genomic DNA of *L. pneumophila* strain Philadelphia-1 as a template. The constructs encompassing residues 1–644 (wild-type; $SetA_{full}$), 14–644 (K577A, K578A, and K580A; $SetA_{full\Delta13}$), 14–474 or 26–474 ($SetA_{NTD}$), 475–644 ($SetA_{linker\cdot CTD}$), and 528–644 ($SetA_{CTD}$) were cloned into expression vector pET-21a(+) (Novagen) between the *Nde*I and *Xho*I restriction enzyme sites. All recombinant proteins were fused with His_6-containing tags at the C-termini (LEHHHHHH). $SetA_{NTD}$ and $SetA_{full}$ were overexpressed in *Escherichia coli* Rosetta 2(DE3) cells, while $SetA_{CTD}$ was overexpressed in *E. coli* Rosetta 2(DE3)pLysS cells. The respective cells were grown at 37°C in Luria Broth culture medium containing 50 µg/ml ampicillin. Protein expression was induced by adding 0.5 mM isopropyl β-D-thiogalactopyranoside and incubating the cells for an additional 20 h at 18°C. The cells were then harvested via centrifugation at 8,600 ×*g* for 10 min at 4°C and subsequently lysed via sonication in ice-cold buffer A (20 mM Tris-HCl [pH 7.9], 500 mM NaCl, and 50 mM imidazole) supplemented with 10% (*v/v*) glycerol and 1 mM phenylmethylsulfonyl fluoride. The crude lysate was then centrifuged at 32,000 ×*g* for 1 h at 4°C to remove cell debris. The supernatant was added to a HiTrap Chelating HP affinity chromatography column (GE Healthcare) pre-equilibrated with buffer A. Proteins were eluted using a linear gradient of buffer A supplemented with 1 M imidazole. The eluted proteins were further purified via gel filtration on a HiLoad 16/60 Superdex 200 prep-grade column (GE Healthcare) pre-equilibrated with buffer B (20 mM Tris-HCl [pH 7.9] and 200 mM NaCl) and were found to be >95% pure as estimated using SDS-PAGE. Fractions containing the recombinant $SetA_{NTD}$ and $SetA_{CTD}$ were pooled and concentrated to 14 mg ml^{-1} and 25 mg ml^{-1} (corresponding to 0.26 mM and 1.8 mM monomer concentrations, respectively) for crystallization using an Amicon Ultra-15 Centrifugal Filter Unit (Millipore). SeMet-labeled $SetA_{NTD}$ and $SetA_{CTD}$ proteins were analyzed using M9 medium. The expression induction and purification of SeMet-labeled SetA proteins were performed as described above.

Small-angle X-ray scattering

To reduce polydispersity and aggregation and to obtain stable samples suitable for SAXS experiments, a stable variant, $SetA_{full\Delta13,}$ was generated by deleting the N-terminal 13 residues predicted to be intrinsically disordered and introducing K577A/K578A/K580A mutations based on the Surface Entropy Reduction (SER) approach (1). $SetA_{full\Delta13}$ proteins were concentrated to 11.3 mg ml^{-1} using a centrifugal concentrator (Millipore), and SAXS data was obtained at the beamline BL-4C of Pohang Light Source, Korea. Before the measurements, the samples were centrifuged for 10 min at 13,000 ×*g* to avoid scattering the precipitated proteins. Buffer B was used to record the reference scattering profiles. Each SAXS profile was collected six times and monitored for radiation damage. To evaluate whether SAXS profile were affected by concentration-dependent oligomerization of aggregation, the samples were serially diluted with buffer C to final concentration of 5.6, 2.8, and 1.4 mg/ml. Each sample was subjected to SAXS measurements and measured six times. Two-dimensional scattering curves were generated from the original scattering images using a homemade program for data from the Pohang Light Source. PRIMUS (2) was used to calculate the radius of gyration (Rg) and D_{max} from the Guinier plot (3). The pair distribution function (P(r)) and Porod volume were calculated using PRIMUS software for each scattering profile. Based on P(r) and the Porod volume, molecular envelopes were calculated using DAMMIF (4). To minimize random errors, 10 different envelopes were generated and the best-fitting model was selected using DAMAVER (5). The SAXS data collection and analysis statistics are presented in Table S1.

Crystallization, data collection, and structure determination

Crystals of both $SetA_{NTD}$ and $SetA_{CTD}$ were grown at 14–23°C by the sitting drop vapor diffusion method using a Mosquito robotic system (TTP Labtech). For the unbound form of $SetA_{NTD}$, 1.4 μl of $SetA_{NTD(14–474)}$ protein solution (14 mg ml^{-1}) was mixed with 0.2 μl of the reservoir solution containing 15% glycerol, 25.5% (*w/v*) polyethylene glycol (PEG)-4,000, 0.2 M lithium sulfate monohydrate, and 0.085 M Tris-HCl (pH 8.5). Next, 0.2 μl of micro crystal seed was added to the drop as an additive. Single crystals of $SetA_{NTD(14–474)}$ appeared within 2 days. In the case of $SetA_{NTD(26–474)}$ complexed with UDP·Glc as a substrate, crystals were obtained from the $SetA_{NTD(26–474)}$ proteins (10 mg ml^{-1}) and a reservoir solution containing 6% (*v/v*) MPD, 14% (*w/v*) PEG-4,000, and 0.1 M sodium/potassium phosphate (pH 6.2), and were soaked for 25 min in the reservoir solution containing 75 mM UDP-Glc and 25% (*v/v*) glycerol. For the UDP-bound $SetA_{NTD}$, diffraction-quality crystals of $SetA_{NTD(26–474)}$ (15 mg ml^{-1}) were grown in a reservoir solution of 18% (*w/v*) PEG-20,000, 0.1 M HEPES (pH 7.0), 5 mM UDP-Glc, and 1 mM $MnCl_2$, giving a product-bound form. For the $SetA_{CTD}$·PI3P structure, $SetA_{CTD(528–644)}$ proteins (12.5 mg ml^{-1}) were incubated with 1 mM inositol 1,3-bisphosphate for 1 h and 1 μl of protein was mixed with 1 μl of a reservoir solution containing 20% (*w/v*) polyacrylic acid-5,100, 0.1 M HEPES (pH 7.0), and 20 mM $MnCl_2$. Crystals of both SeMet-labeled proteins, $SetA_{NTD(14–474)}$ and $SetA_{CTD(528–644)}$ were obtained under conditions identical to those of the corresponding native crystals.

X-ray diffraction data from $SetA_{NTD}$ and $SetA_{CTD}$ were collected at beamlines BL-5C, BL-7A, and BL-11C of Pohang Light Source, Korea, and beamline BL44XU of Spring-8, Japan. Data were processed and scaled using the program suite HKL2000 (6) or XDSGUI (https://strucbio.biologie.uni-konstanz.de/xdswiki/index.php/XDSGUI). The crystals were flash-frozen under a stream of nitrogen gas at 100 K. Since SeMet-labeled crystals of $SetA_{NTD·14–474}$ diffracted poorly to 4 Å only, they were soaked in a Paratone oil as a cryoprotectant, giving a more improved diffraction resolution at 2.85 Å and 2.54 Å. Native crystals of $SetA_{NTD(26–474)}$ and SeMet-labeled crystals of $SetA_{CTD·528–644}$ were then transferred to the corresponding reservoir buffer containing 20–25% (v/v) glycerol for cryoprotection. Tables S2 and S3 present the data collection statistics.

For the phase determination of SeMet-labeled $SetA_{NTD(26–474)}$ and $SetA_{CTD(528–644)}$, single-wavelength anomalous diffraction (SAD) experiments were performed. AUTOSOL of the PHENIX software package (7) identified 14 and 8 Se sites in an asymmetric unit, respectively. The phases were further improved by building an initial model using the automatic model-building program RESOLVE (8). The models of SeMet-labeled $SetA_{NTD}$ and $SetA_{CTD}$ were constructed using iterative cycles of model-building with Coot (9) and refinement with Refmac5 in the CCP4 suite (10). A total of 5% of the data was randomly chosen as the test set for calculating the R_{free} (11). The structures of $SetA_{NTD}$·UDP-Glc, $SetA_{NTD}$·UDP, and $SetA_{CTD}$·PI3P were determined from their native crystals using molecular replacement with the program MolRep (Vagin and Teplyakov, 2010) using the refined models of SeMet-labeled $SetA_{NTD}$ and $SetA_{CTD}$. The stereochemistry of the refined models was evaluated using MolProbity software (12). Tables S2 and S3 present the phasing and model refinement statistics, respectively.

SEC-MALS

Size-exclusion chromatography with multi-angle light scattering (SEC-MALS) experiments for $SetA_{full}$, $SetA_{NTD(14–474)}$, $SetA_{linker·CTD}$, and $SetA_{CTD}$ were performed using an FPLC system (GE Healthcare) connected to a Wyatt DAWN HELOEOS II MALS instrument and a Wyatt Optilab T-rEX differential refractometer (Wyatt Technology Corporation, Santa Barbara, CA). A Superdex 200 10/300 GL (GE Healthcare) gel filtration column pre-equilibrated with buffer B (20 mM Tris-HCl [pH 7.9] and 200 mM NaCl) was normalized using ovalbumin. Protein samples (1 mg ml^{-1} and 7.3 mg ml^{-1}; volume of 100 μl) were injected at a flow rate of 0.5 ml min^{-1} at 25°C. Data were analyzed using the Zimm model for fitting static light scattering data and were graphed using EASI graph with a UV peak in ASTRA 6

software (Wyatt).

In vitro glucosylation of synthetic peptides and mass spectrometry

Ultrafiltration

The $SetA_{NTD}$ protein (16.7 μM) and hRab1-derived peptides corresponding to residues 72–79 (wild-type, S78P or R74A; 1.5 mM) were incubated at 37°C for 14 h in a buffer (total 20 μl) containing 20 mM Tris-HCl (pH 7.9), 150 mM NaCl, 1 mM $MnCl_2$, 1 mM $MgCl_2$, and 2.5 mM UDP-Glc. As a negative control, we monitored glucosylation in the absence of SetA. The reaction mixture and standard hRab1 peptides were centrifuged using Amicon Ultra-4 3 K centrifugal filter devices (Millipore, Billerica, MA, USA), according to the manufacturer's instructions. The flow-through liquid was evaporated to dryness using a concentrator 5301 (Eppendorf, Hamburg, Germany) and resuspended in 0.1% formic acid (FA) before LC-MS analysis.

LC-MS/MS analysis

The eluted peptides were analyzed using a Q Exactive^{TM} HF-X hybrid quadrupole-Orbitrap mass spectrometer (Thermo Fisher Scientific) coupled to an Ultimate 3000 RSLCnano system (Thermo Fisher Scientific). The peptides were loaded onto a trap column (100 μm × 2 cm) packed with Acclaim PepMap100 C18 resin. The loaded peptides were then eluted with a linear gradient from 7 to 28% solvent B (0.1% FA in acetonitrile) for 90 min at a flow rate of 300 nl min^{-1}. The eluted peptides, separated by an analytical column (EASY-Spray column, 75 μm × 50 cm, Thermo Fisher Scientific), were sprayed into a nano-ESI source at an electrospray voltage of 2.5 kV. The Q Exactive HF-X Orbitrap mass analyzer was operated using the top 20 data-dependent methods. Full MS scans were acquired over the m/z range of 350–1800, with a mass resolution of 60,000 (at m/z 200). The automatic gain control target value was 3.00×10^6. The twenty most intense peaks with charge states ≥2 were fragmented in the higher-energy collisional dissociation collision cell with a normalized collision energy of 28, and tandem mass spectra were acquired in an Orbitrap mass analyzer with a mass resolution of 15,000 at m/z 200.

UDP-Glc hydrolase activity assay

The relative hydrolysis activities of $SetA_{full}$ wild-type or mutants (W36A, R121A, Y132A, D134A/D136A, D136A, and K210A) at a concentration of 4.58 μM were studied by measuring the release of free UDP using an ADP Quest Assay kit (DiscoverRx) according to the manufacturer’s protocol. The assay was performed after incubation at 37°C for 30 min in a 384-well black assay plate with a total volume of 20 μl per well. Experiments from four independent assays were performed with a UDP-Glc concentration of 300 μM in a buffer consisting of 20 mM Tris (pH 7.9), 100 mM NaCl, 1 mM $MnCl_2$, and 1 mM $MgCl_2$.

SPR analysis

The kinetics and affinity of $SetA_{NTD}$ for the Rab1 protein and Rab1-derived peptides were assessed using a Reichert SR7500 SPR dual-channel instrument (Reichert, Depew, NY). Briefly, the $SetA_{NTD}$ proteins purified in buffer C (20 mM HEPES [pH 8.0], 150 mM NaCl, and 1 mM TCEP) were immobilized using standard amino coupling at a rate of 30 μl min^{-1} on a carboxymethyl dextran hydrogel surface sensor chip (Reichert, Depew, NY) until saturation was achieved. Running buffer D (10 mM HEPES [pH 7.4], 150 mM NaCl, 3 mM EGTA, and 0.005% [*v/v*] Tween-20) was used in all SPR experiments. The SPR experiments were performed at 20°C. Nine concentrations (3.90, 7.81, 15.6, 31.3, 62.5, 125, 250, 500, and 1,000 μM) of each of the Rab1 peptides 1, 2, 3 and six concentrations (1.95, 3.90, 7.81, 15.6, 31.3, and 62.5 μM) of Rab1 wild-type protein ($Rab1_{WT}$) were prepared in buffer C. Serially diluted peptides and protein solutions were injected over the $SetA_{NTD}$ chip at a rate of 30 μl min^{-1} for 5 min for the association analyses. Subsequently, running buffer D was applied over the chip for an additional 6 min (30 μl min^{-1}) for dissociation analyses. The chip was then

regenerated with 5 mM NaOH. Binding was detected as a change in the refractive index at the chip surface as measured in response units (RU). A reference flow cell was used as a positive control to record the response to bovine serum albumin (BSA), which was subtracted from each sample. SPR data were fitted using Scrubber2 software (13).

To study the interaction between $SetA_{NTD}$ and the two Rab1 mutants ($Rab1_{S25N}$ and $Rab1_{Q70L}$), SPR experiments were performed using a Biacore T200 instrument. Briefly, $SetA_{NTD}$ was immobilized onto a CM5 chip (GE Healthcare, Little Chalfont, UK) surface via amine coupling until 1,000 RU was reached. All SPR experiments were performed at 20°C with running buffer D. $Rab1_{S25N}$ and $Rab1_{Q70L}$ proteins were purified using a gel filtration column pre-equilibrated with buffer C supplemented with 5 mM GDP and GppNHp, respectively. Seral dilutions (2.34 to 150 μM) of the Rab1 mutants as analytes were prepared just before injection. The analyte solutions were injected at a flow rate of 30 μl min^{-1} to monitor association for 2 min, and then for an additional 6 min for dissociation analyses. After 5 min, the chip was washed with 5 mM NaOH to remove any unattached analytes. SPR sensorgram monitoring of the control protein was performed in response to BSA in the measuring flow cells. Finally, SPR data were fitted using Biacore T200 evaluation software (version 3.0).

Microscale thermophoresis (MST)

The binding affinity between $SetA_{NTD}$ and UDP-Glc was determined using a Monolith NT.115 instrument (NanoTemper) at 22°C and measured at 30% LED and 40% MST power with a 30 s laser on time and 5 s laser off time. $SetA_{NTD}$ proteins were prepared at a final concentration of 200 nM in a buffer consisting of 20 mM Tris-HCl (pH 7.9), 150 mM NaCl, and 1 mM TCEP. His_6-tagged $SetA_{NTD}$ proteins were labeled using 50 nM Red-Tris NTA dye (NanoTemper), and the concentration of the labeled protein was maintained at 200 nM. Next, 10 μl of the labeled $SetA_{NTD}$ proteins were mixed with 10 μl of the UDP-Glc solutions at various concentrations (from 7.63 μM to 125 mM) in a buffer containing 20 mM Tris-HCl (pH 7.9), 150 mM NaCl, and 0.05% Tween-20, and were incubated for 10 min at room temperature before the MST assay. The K_D was calculated by fitting a standard binding curve to the average of three independent dilution series using the MO Affinity Analysis v2.3 software (NanoTemper).

Protein-lipid overlay assay

To test the direct binding of $SetA_{CTD}$ to lipids, experiments were carried out with the purified $SetA_{CTD}$ wild-type or double mutants (K577Q/K578Q, K595Q/K598Q, and R605Q/K621Q) using PIP Strips^{TM} (spotted with 100 pmol of 15 different lipids) or a Custom PI3P Array (spotted with seven different concentrations of 1.56–100 pmol of PI3P) that were purchased from Echelon Biosciences. After blocking, the strips were incubated with 1200 pmol of $SetA_{CTD}$ wild-type or mutants for 1 h in 1% BSA (BOVOGEN) in Tris-buffered saline with 0.1% Tween-20 (TBS-T), washed with TBS-T, and incubated with anti-His HRP (sc-8036 HRP; Santa Cruz; 1:1000 dilution). The membranes were then visualized using a FUSION© Solo 5 instrument (Vilber Lourmat). Quantification of the protein-lipid overlay assays was performed in two independent experiments.

Cell proliferation

HEK293 cells were plated at a density of $1x10^5$ cells per well in 6-well plates in triplicate. Plasmid DNA (pCMV-3Tag-1(Control) or pCMV-3Tag-SetA(SetA) was transfected using Lipofectamine 3000 reagent the next day. On each of two consecutive days, cells were fixed with 4% PFA for 10 minutes at RT, treated with 0.5% Triton X-100 for 5 minutes at RT, and stained with 0.5 μg/ml DAPI for 10 minutes at RT. Cell growth was measured using a Cytation 3 instrument (BioTek).

Immunofluorescence

The cells were plated onto coverslips and fixed with 4% paraformaldehyde (PFA) for 10 minutes and

permeabilized with 0.5% Triton X-100 for 5 minutes at RT. The cells were blocked with 3% BSA in PBS for 30 minutes at RT and incubated with primary antibodies at 4°C overnight. The primary antibodies used were as follows: anti-Flag(Sigma, F1804 and Millipore, F7425), anti-GM130 (BD Biosciences, 610823), anti-Calnexin (Abcam, ab22595) and anti-Rab1(Santa cruz, sc-515308). After PBS washes, the cells were incubated with secondary antibodies (anti-mouse A488 and A594; anti-rabbit A488 and A594; Life Technologies) for 2 hours at RT. After the nuclei were stained with 0.5 μg/ml 4',6-diamidino-2-phenylindole (DAPI), the coverslips were mounted with ProLong Gold Antifade Reagent (Life Technologies), and the slides were observed using a confocal microscope (LSM780 or LSM880 Airyscan, Carl Zeiss). The image was processed using ZEN blue 3.1 software (Carl Zeiss).

Docking and complex model generation

Computational docking was performed using the Glide module in the Schrödinger software suite (14). The receptor was prepared using the $SetA_{NTD}$·UDP·Glc crystal structure. As the substrate, a synthetic Rab1 peptide corresponding to residues 72–79 (RFRTITSS), which are predicted to form a loop in GDP-bound Rab1, was used. Flexible peptide docking was then conducted to identify plausible binding orientations within the expanded active site groove. The resulting high-scoring docking pose, which positions the Rab1 glycosylation target residue in a catalytically plausible orientation toward the GTase active site, served as an anchoring reference for generating the full protein complex. The GDP-bound Rab1 structure (PDB 2FOL) was superposed onto this docked peptide pose to reconstruct the complete SetA-Rab1 assembly. The final resulting model was visually inspected using PyMOL to confirm the absence of steric clashes.

SI References

1. L. Goldschmidt, D. R. Cooper, Z. S. Derewenda, D. Eisenberg, Toward rational protein crystallization: A Web server for the design of crystallizable protein variants. *Protein Sci* **16**, 1569-1576 (2007).
2. P. V. V. Konarev, V. V.; Sokolova, A. V.; Koch, M. H. J.; Svergun, D. I., PRIMUS: a Windows PC-based system for small-angle scattering data analysis. *Journal of Applied Crystallography* **36**, 1277-1282 (2003).
3. D. I. Svergun, Determination of the regularization parameter in indirect-transform methods using perceptual criteria. *Journal of Applied Crystallography* **25**, 495-503 (1992).
4. D. Franke, D. I. Svergun, DAMMIF, a program for rapid ab-initio shape determination in small-angle scattering. *J Appl Crystallogr* **42**, 342-346 (2009).
5. V. V. S. Volkov, D. I., Uniqueness of ab initio shape determination in small-angle scattering. *Journal of Applied Crystallography* **36**, 860-864 (2003).
6. Z. Otwinowski, W. Minor, Processing of X-ray diffraction data collected in oscillation mode. *Methods in enzymology* **276**, 307-326 (1997).
7. P. D. Adams *et al.*, PHENIX: a comprehensive Python-based system for macromolecular structure solution. *Acta Crystallogr D Biol Crystallogr* **66**, 213-221 (2010).
8. T. C. Terwilliger, Automated main-chain model building by template matching and iterative fragment extension. *Acta Crystallogr D Biol Crystallogr* **59**, 38-44 (2003).
9. P. Emsley, B. Lohkamp, W. G. Scott, K. Cowtan, Features and development of Coot. *Acta Crystallogr D Biol Crystallogr* **66**, 486-501 (2010).
10. G. N. Murshudov, A. A. Vagin, E. J. Dodson, Refinement of macromolecular structures by the maximum-likelihood method. *Acta Crystallogr D Biol Crystallogr* **53**, 240-255 (1997).
11. A. T. Brunger, Free R value: a novel statistical quantity for assessing the accuracy of crystal structures. *Nature* **355**, 472-475 (1992).

12. V. B. Chen *et al.*, MolProbity: all-atom structure validation for macromolecular crystallography. *Acta Crystallogr D Biol Crystallogr* **66**, 12-21 (2010).
13. Y. Wei, R. A. Latour, Determination of the Adsorption Free Energy for Peptide−Surface Interactions by SPR Spectroscopy. *Langmuir* **24**, 6721-6729 (2008).
14. R. A. Friesner *et al.*, Extra precision glide: docking and scoring incorporating a model of hydrophobic enclosure for protein-ligand complexes. *Journal of medicinal chemistry* **49**, 6177-6196 (2006).
